# Reducing antimicrobial use in food animals: Consider user fees and regulatory caps on veterinary use

**DOI:** 10.1126/science.aao1495

**Published:** 2017-09-29

**Authors:** Thomas P. Van Boeckel, Emma E. Glennon, Dora Chen, Marius Gilbert, Timothy P. Robinson, Bryan T Grenfell, Simon A. Levin, Sebastian Bonhoeffer, Ramanan Laxminarayan

**Affiliations:** 1 Institute of Integrative Biology, ETH Zurich, Zurich 8006, Switzerland; 2 Center for Disease Dynamics, Economics and Policy, Washington, DC 20005, USA; 3 Department of Veterinary Medicine, University of Cambridge, Cambridge CB3 0ES, UK; 4 Department of Ecology and Evolutionary Biology, Princeton University, Princeton, NJ 08544, USA; 5 Université Libre de Bruxelles, Brussels 1050, Belgium; 6 Fonds National de la Recherche Scientifique, Brussels 1050, Belgium; 7 International Livestock Research Institute, Nairobi 00100, Kenya; 8 Food and Agriculture Organization of the United Nations, Rome 00153, Italy; 9 Fogarty International Center, National Institutes of Health, Bethesda, MD 20892, USA; 10 Princeton Environmental Institute, Princeton University, Princeton, NJ 08544, USA

The large and expanding use of antimicrobials in livestock, a consequence of growing global demand for animal protein, is of considerable concern in light of the threat of antimicrobial resistance (AMR). Use of antimicrobials in animals has been linked to drug-resistant infections in animals (*1*) and humans (*2*). In September 2016, the United Nations (UN) General Assembly recognized the inappropriate use of antimicrobials in animals as a leading cause of rising AMR. In September 2018, the interagency group established by the UN Secretary General will report on progress in the global response to AMR, including antimicrobial consumption in animals. We provide a baseline to monitor efforts to reduce antimicrobial use and assess how three global policies might curb antimicrobial consumption in food animal production: (i) enforcing global regulations to cap antimicrobial use, (ii) adherence to nutritional guidelines leading to reduced meat consumption, and (iii) imposing a global user fee on veterinary antimicrobial use.

The rise of AMR in zoonotic pathogens, including to last-resort drugs such as colistin (*3*), is an important challenge for human medicine because it can lead to untreatable infections. Evidence linking AMR between animals and humans is particularly strong for common foodborne pathogens resistant to quinolones, such as *Campylobacter* spp. and *Salmonella* spp. (*4*). AMR is also a threat to the livestock sector and thus to the livelihoods of millions who raise animals for subsistence (*5*).

The primary driver for the accumulation of harmful resistance genes in the animal reservoir is the large quantity of antimicrobials used in animal production (*6*). Antimicrobial use in livestock, which in many countries outweighs human consumption (*7*), is primarily associated with the routine use of antimicrobials as growth promoters or their inappropriate use as low-cost substitutes for hygiene measures that could otherwise prevent infections in livestock.

In Europe, regulations have been the principal instrument to limit antimicrobial use in animal production. In the United States, consumer preferences have driven companies to reduce antimicrobial use in animals, although the impact on livestock rearing practices is still nascent (*8*). Some European countries maintain highly productive livestock sectors while using less than half the current global average amount of antimicrobial per kilogram of animal (50 mg/kg). Therefore, this threshold has been proposed as a potential target for global regulations on veterinary antimicrobial use (*9*). However, the impact that such policies would have on the global consumption of antimicrobials has yet to be quantified.

A second solution to reduce antimicrobial consumption in animal production may be to promote low-animal-protein diets: China has recently revised downward its nutritional guidelines for meat intake to 40 to 70 g/day (*10*), which is approximately half the current consumption level in the country. If followed, this measure could have an indirect but substantial impact on the global consumption of veterinary antimicrobials. A third solution to cut antimicrobial use would be to charge a user fee, paid by veterinary drug users, on sales of antimicrobials for nonhuman use (*11*). This approach has recently received support from the World Bank (*12*) on the basis that the associated revenues could be injected into a global fund to stimulate discovery of new antimicrobials and support efforts to preserve existing drugs (*13*). Without further analysis, however, it is unclear whether a user fee policy could achieve a meaningful reduction in the global consumption of veterinary antimicrobials, let alone generate sufficient revenues to support improved livestock rearing practices or the development of new drugs, vaccines, and diagnostics.

## GLOBAL TRENDS

Veterinary antimicrobial sales volumes were obtained via public records for 38 countries and self-governing dependencies and estimated for 190 more (supplementary materials). In 2013, the global consumption of all antimicrobials in food animals was estimated at 131,109 tons [95% confidence interval (CI) (100,812 to 190,492 tons)] and is projected to reach 200,235 tons [95% CI (150,848 to 297,034 tons)] by 2030. Consumption levels varied considerably between countries, ranging from 8 mg/population correction unit (PCU) (a kilogram of animal product) in Norway to 318 mg/PCU in China (see fig. S1). As the largest consumer of veterinary antimicrobials, both in relative (per PCU) and in absolute terms, China has an important leadership role with regard to its response to AMR and has already set precedents in phasing out drugs that are last resorts for human infections but are still in use in Europe in animal husbandry.

In relative terms, humans and animals use comparable amounts of antimicrobials [118 mg/PCU and 133 mg/kg, respectively (*14*)], but given that the biomass of animals raised for food exceeds by far the biomass of humans, new resistant mutations are more likely to arise in animals. Furthermore, a central distinction between animals and humans is the purpose of antimicrobial use. Unlike in humans, antimicrobial use in animals is primarily intended for growth promotion and mass prophylaxis. These uses are often administered both through feed, directly targeting the gut, and in low-dose patterns that promote the evolution of resistance (*15*). These factors suggest that the food animal reservoir is a greater source of resistance genes than humans. However, the subsequent spread of those genes to humans follows complex pathways, and recent work has highlighted that curtailing antimicrobial use in animals alone will not suffice to contain AMR in humans (*16*).

**Figure:**
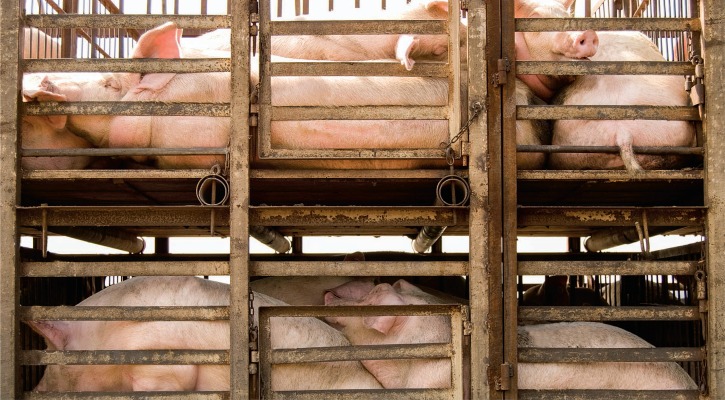
Pigs in cages, Quanzhou, China. As the largest consumer of veterinary antimicrobials, China is critical for combating antimicrobial resistance (AMR).

## GLOBAL SOLUTIONS

The use of antimicrobials in food animals could be reduced by 2030 between *9* and 80% with effective policies compared with a business-as-usual target (BAU) of continued growth of the livestock sector with current levels of antimicrobial use (see the graph). This could be achieved either by reducing the quantity of antimicrobial used per animal (targets 1 and 3) or the number of animals that we raise for food (target 2).

*Regulations.* A global regulation putting a cap of 50 mg of antimicrobials per PCU per year, the current global average amount, could reduce total consumption by 64% (target 1A). If only countries of the Organization for Economic Cooperation and Development (OECD) and China were to adopt this regulation, the global consumption in 2030 would already be reduced by 60% (target 1B). In the short term, target 1B may be preferred because it would have substantial impact on global consumption without targeting vulnerable farmers in low- and middle-income countries (LMICs) who rely on the ability to treat livestock for subsistence (*17*). In some high-income countries, regulatory approaches have achieved substantial reduction in antimicrobial use within a few years and at moderate costs. However, in LMICs, the cost of setting up surveillance systems is a barrier to enforcement, and our findings are contingent on enforceability.

*Meat consumption.* Limiting meat intake worldwide to 40 g/day—the equivalent of one standard fast-food burger per person— could reduce global consumption of antimicrobials in food animals by 66% (target 2A). This reduction is comparable with what could be achieved through regulations targeting antimicrobial use (targets 1A and 1B). In comparison, meat consumption in the United States currently averages 260 g/ day (OECD 2015). In this context, and given increasing appetites for meat in emerging economies, it seems unlikely that antimicrobial use in food animals could be reduced substantially through voluntary adherence to such drastic changes in dietary habits. Under a more realistic global cap of 165 g meat/day (projected EU average in 2030), global consumption of antimicrobials could be reduced by 22% (target 2B). Reduced meat consumption could thus have substantial benefits on AMR as well as other environmental and human health issues.

*User fees.* Imposing a user fee of 50% of the current price on veterinary antimicrobials could reduce global consumption by 31% (target 3C). More important, such a policy would also generate yearly revenues between US$ 1.7 billion and 4.6 billion (Protocol S4). In comparison, the level of investment necessary for the development of one new antimicrobial compound is typically US$ 1 billion (*18*). Alternative rates of 10 or 100% for the user fee would reduce the global consumption by 9 and 46%, generating revenues of US$ 0.4 billion to 1.2 billion and US$ 2.8 billion to 7.5 billion, respectively. Concretely, the fee could be applied at the point of manufacture or wholesale purchase for imported products. The advantages of this implementation are twofold. First, given the limited number of drug manufacturers, enforcement would require only limited resources. Second, manufacturers are more likely than veterinarians to keep records of volumes traded, especially in countries where drugs are used without prescription. However, because user fees could be passed on to individual farmers, these could also have adverse effects if not accompanied by other measures to reduce the need for antimicrobials in food production. Here, we identify that demand for veterinary antimicrobials is on average more elastic in LMICs (Protocol S4), with the notable exception of China, where demand was inelastic because of increased reliance on antimicrobials for food production. LMICs could therefore be disproportionally affected by a user fee.

## COMPARISON AND LIMITATIONS

The solutions presented in this analysis are not mutually exclusive; if considered in combinations, regulatory caps, user fees, and reductions in meat intake could potentially reduce global consumption of antimicrobials in animals by up to 80%. However, implementation of those policies should account for differences across income groups. We show that a global user fee policy could circumvent the limitations inherent to regulatory approaches while still achieving a meaningful reduction in antimicrobial use (31%).

Unlike regulations that may be virtually impossible to enforce in LMICs, a user fee policy could be applied immediately, without waiting for costly surveillance networks to put in place. In LMICs, large livestock producers could follow the example from European countries, where drastic reductions in antimicrobial consumption could have potential long-term benefits. In compensation for the reduction in antimicrobial use in LMICs, major investments will be needed to improve farm hygiene and expand veterinary services. We show that these could be partly financed with the revenues of the user fee policy through a global fund. In parallel, national programs should also ensure that antimicrobials used for treatment by smallholders remain affordable so that a global user fee doesn’t become an obstacle for livestock- driven economic development.

In the long run, this transition to low antimicrobial use could benefit all countries: Phasing out growth that promotes antimicrobials will likely have limited impact on food production (*19*) but would reduce the risk of emergence of pathogens resistant to lastresort drugs (*3*). Reducing antimicrobial use may also benefit LMICs to secure export markets where customers express preferences for products obtained without antimicrobials (*8*) and restriction on antimicrobial use may apply as part of trade agreements.

Our findings are subject to limitations. For example, although more countries (including LMICs) have reported sales of antimicrobials for this estimate compared with 2010 (*20*), information on sales broken down by species and by classes of compounds is still limited. As a result, consumption in nonreporting countries can only be estimated through extrapolations. In addition, available information on antimicrobial prices prevents a more advanced economic analysis on the impact of user fees than presented in this study. Unlike for human medicine, there is currently no global database (public or private) on veterinary antimicrobial sales accessible to the public health community. Although present data are limited, outlining current knowledge allows inferences to be made about the relative impact of different policies to curb antimicrobial use. Our findings suggest that imposing a user fee on veterinary antimicrobials is a plausible policy option to achieve meaningful reductions in antimicrobial use in the short term while simultaneously raising funds to improve farming practices that will benefit the long-term viability of the livestock industry.

**Figure:**
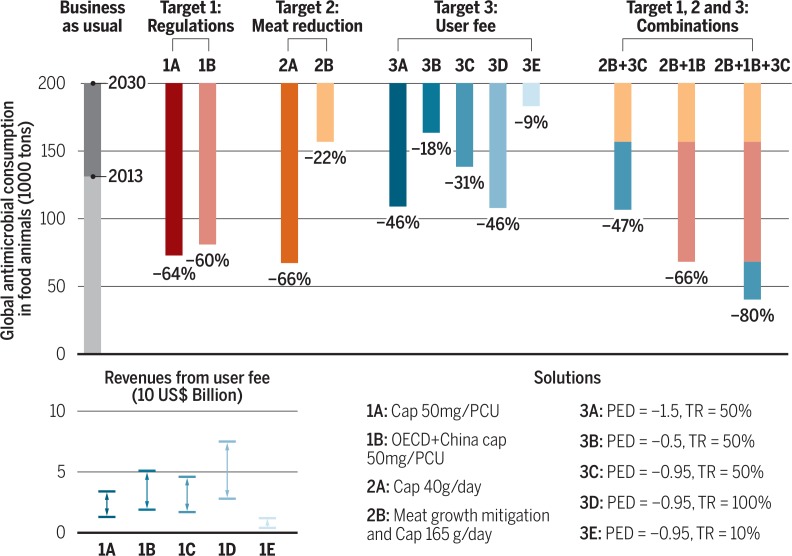
Antimicrobial consumption in food animals by 2030 Business as usual and intervention policies are shown. Revenue ranges are estimated for different fee rates (TR) and price elasticities of demand (PED). For 3C, 3D, and 3E, PEDs are derived from time series of imports of veterinary antimicrobials in each country (Protocol S4); the global average PED was -0.95. See supplementary materials for discussions of uncertainty in all estimates shown in figures. PCU, population correction unit.

## SUPPLEMENTARY MATERIALS

www.sciencemag.org/content/357/6358/1350/suppl/DC1

## Antimicrobial consumption in food animals by 2030

Business as usual and intervention policies are shown. Revenue ranges are estimated for different fee rates (TR) and price elasticities of demand (PED). For 3C, 3D, and 3E, PEDs are derived from time series of imports of veterinary antimicrobials in each country (Protocol S4); the global average PED was -0.95. See supplementary materials for discussions of uncertainty in all estimates shown in figures. PCU, population correction unit.
