# Supplementary material for: Reducing antimicrobial use in food animals: Consider user fees and regulatory caps on veterinary use
Source: Science. 2017 Sep 29;351(6358):1350–2. doi: 10.1126/science.aao1495 (PMC6510296; doi:10.1126/science.aao1495)
Supplement: Supplementary file 1 [file Science-357-1350_S1.pdf]

Science AAAS

www.sciencemag.org/content/357/6358/1350/suppl/DC1

Supplementary Material for

Reducing antimicrobial use in food animals

Thomas P. Van Boeckel, Emma E. Glennon, Dora Chen, Marius Gilbert, Timothy P. Robinson, Bryan T Grenfell, Simon A. Levin, Sebastian Bonhoeffer, Ramanan Laxminarayan

Email: ramanan@cddep.org

Published 29 September 2017, *Science* **357**, 1350 (2017)
DOI: 10.1126/science.aao1495

This PDF file includes:

Materials and Methods
Figs. S1 to S9
Tables S1 to S3
References

Materials and Methods

Data used in this paper can be found at

https://www.dropbox.com/sh/9k4oojy1fu8hsbw/AABoobcb_8GPM48BHpjsCQgLa?dl=0

Protocol S1. Antimicrobials sales data

We conducted systematic online search for sales data on veterinary antimicrobials from government sources and scientific publications. The procedure for the online search is identical to Van Boeckel et al 2015 (*1*), but novel data sources were incorporated in this study (Table S1). Reports of antimicrobials sales volumes (Tons) were obtained for 38 countries (Fig S1). The countries where antimicrobial sales could be obtained represent 47% of the estimated global biomass of livestock standing and slaughtered each year (FAOSTAT 2013). In this study, only medically important antimicrobials were considered; ionophores and coccidiostats were excluded from the analysis. Reports of sales were obtained using the year 2013 as reference, or the most recent year for which data broken down by species or classes of compounds were available. In 17 countries, sales were broken down by at least two groups of animals. If two countries have the same relative proportion for different types of livestock, their respective consumption can nonetheless differ because of local disease prevalence, climate, market, legislations and implantation of drug manufacturers. In order to account for these differences we adapted the methodology from Van Boeckel et al. 2015, to estimate the consumption of antimicrobials by classes of compounds (see Statistical Procedure) and by species.

All bird species were pooled in a single poultry category (chicken and ducks) as well as small ruminants (sheep and goats). For New Zealand reports of sales of antimicrobial were pooled together for horse and sheep. We made the assumption that antimicrobial use in horse could be neglected compared with sheep, particularly given the large size of this sector in New Zealand and have assumed that sheep represent the total consumption reported. In Belgium, antibiotic sales per species were obtained for 2009 and adjusted for 2013 using the overall volume of antimicrobial. We checked for systematic correlation (drug used in combination treatment) or inverse correlation (substitution of drugs) for all the non-tetracycline drugs (Pearson coefficients <0.6). In France antimicrobial consumption in cattle and small ruminants was reported in a single pooled category. We distributed the total using a ratio of 1/3.575, which corresponds the mean ratio in countries that had reported animal specific data for both sheep and cattle.

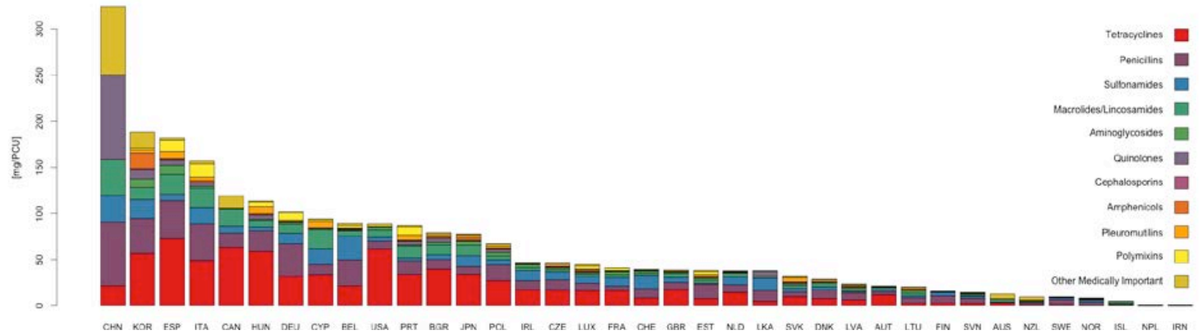

Fig S1. Reported sales of veterinary antimicrobials. Consumption levels in milligrams per population correction unit for 10 classes of compounds (Table S1).

<u>Protocol S2. Livestock Census</u>

This study includes four categories of animals: chicken, cattle, pigs and small ruminants (sheep and goats, hereafter referred as 'sheep'), which together account for the overwhelming majority of terrestrial animals raised for food (*2*). Sheep are important sources of animal-protein in Africa and Asia –two regions where meat consumption is growing rapidly. In each country (or self-governing dependency, hereafter referred to as 'Country'), we estimated the total biomass of animals using population correction units (PCU). PCU represent the number of kilograms of animal alive or slaughtered each year. This metric is used to standardize estimates of biomass between countries that have variations in average weight of the animals or number of production cycles per year. Livestock census disaggregated by production systems (extensive vs intensive) were obtained from Robinson et al (*3*) and projected for the year 2013.

The total PCUs in any country (or pixel) for livestock type k in the production system s were defined as follows:

$$PCU_{k,s} = An_{\,k,s} \cdot (1 + n_{k,s}) \cdot \left(\frac{Y_k}{R_{\frac{CW}{LW},k}} \right)$$

where $An_{\,k,s}$ is the stock of the animal of type 'k' raised in the production systems 's' (extensive or intensive) that is alive in a country/pixel, $n_{k,s}$ is the number of production cycles from animals 'k' in each production system 's' in the corresponding country, Y is the quantity of meat per animal obtained for each country, and $R_{CW/LW}$ is the killing-out percentage (or dressing percentage)—that is, the ratio of the carcass weight that result from partial butchering and removing all the less desirable parts (organs, tail, feet) to live weight of an animal—obtained from literature estimates (*4*). The last term of this equation can be interpreted as the animal weight reconstructed from country-specific productivity figures. All parameters were obtained from FAOSTAT for the year 2013.

Protocol S3. Extrapolation of Antimicrobial Consumption

We used a five step statistical procedure adapted from Van Boeckel et al (*1*), to extrapolate antimicrobial consumption from countries reporting sales (n=38) to all other countries. The methodology was adapted to include sheep, and 10 different classes of compounds (Protocol S1). The objective of this procedure is to calculate coefficients for the consumption for each class of antimicrobials (c=10) in each country (i=228) for each group of livestock species (k=4) and in each production system (s = extensive *or* intensive).

Step 1: Consumption Intensity We used a multivariate regression model including the 38 reporting countries to establish a statistical relationship between the overall antimicrobial consumption and the PCU of chicken and pigs raised in intensive systems. This relationship was subsequently used to predict overall intensity of antimicrobial use in intensive systems (mg/PCU) in all other countries (n=190).

Step 2: Tetracyclines use vs Consumption Intensity. We used a univariate regression model including all reporting countries to establish a relationship between the consumption intensity in intensive systems (mg/PCU_{int}) and the percentage tetracycline π_{TET} as part of the total antimicrobial consumption (Fig. S2). This relationship was subsequently used to predict the proportion of tetracycline used in each country according to its consumption intensity (step 1). This reflected the fact that tetracyclines are proportionally overused in high-consuming countries. The respective proportions of non-tetracycline compound were derived from the mean proportions of each of those compounds in all reporting countries (no significant relationship between intensity and overuse of other compounds was found). All proportions were adjusted to sum up to one, and replaced by original data for the reporting countries.

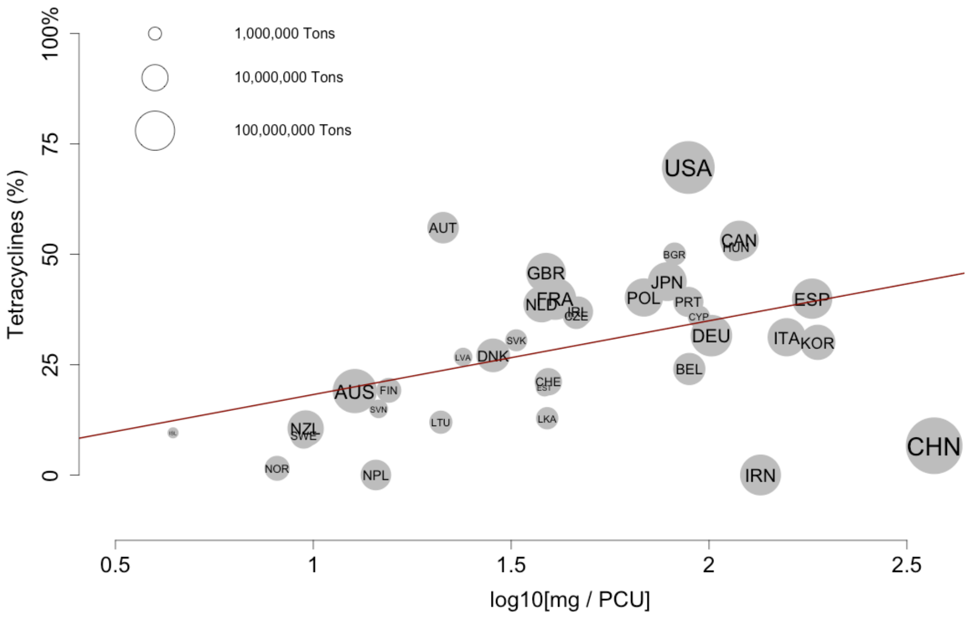

Fig S2. Share of tetracycline in a country vs antimicrobial consumption per kilogram of animal. Circles are proportional to the log10 of the PCU in each country.

Step 3: Species-specific consumption. We used countries where species-specific data could be obtained to compute species-specific relative proportions for different compounds ($\pi_{k,c}$). For countries where consumption by species was provided by class of compound (SCB), these values were used directly to compute coefficients of consumption for intensive system. For countries where species consumption levels were aggregated across compounds (SB), this overall volume was disaggregated in 10 $\alpha_{c,i,k,s}$ coefficients, according to the mean proportions $\pi_{c,k}$ computed in SCB countries. Finally, in every country the value of the species-specific consumption for tetracycline ($\pi_{TET,k}$), was adjusted to reflect the increased tetracycline consumption in high-consuming countries (Step 2) and the consumption for other compounds was modulated accordingly. Finally, in the countries were species-specific coefficient were available from the collected data the $\alpha_{c,i,k,s}$ coefficients were scaled to match the reported values (Fig S8).

Step 4: Extensive vs intensive production systems. In agreement with Van Boeckel et al. 2015 (*1*), we assumed that intensive systems consume on average four times the amount extensive systems per kilogram of livestock produced. Finally, estimates of the global consumption by species, production systems, and countries were obtained by multiplying the coefficients of consumption by the number of PCU in each corresponding livestock system. Confidence intervals on the total consumption were calculated as 1.96 times the standard deviation associated with the coefficients of consumption per species (step 3). Negative predictions for the lower bound were ceiled to zero. Thus the global consumption of antimicrobial and its associated confidence interval (95% C.I.) was estimated as,

$$Global\ Consumption = \sum_{j}^{228}\sum_{c}^{10}\sum_{k}^{4}\sum_{s}^{2}(\alpha_{c,j,k,s} \pm 1.96 \cdot sd(\alpha_{c,j,k,s})) \cdot PCU_{2013,c,j,k,s}$$

where $sd(\alpha_{c,j,k,s})$, is the standard deviation on the estimated coefficient from step 3. In 2013, the global consumption of antimicrobials in food animals was 131,109 tons (95% C.I. [100,812 - 190,492]). Based on future trends for consumption of livestock products (see next section), the global consumption of antimicrobials in food animals is projected to reach 200,235 tons in 2030 (95% CI [150,848 - 297,034]). Fig S6 shows the breakdown of the global antimicrobial consumption by drug-class, species and production systems.

Protocol S4. Projections for antimicrobial use in 2030

We projected the future consumption of antimicrobials under four scenario corresponding to different targets for antimicrobial use.

***Business as usual target (BAU*).** Current levels of antimicrobials consumption were projected in 2030. First, trends for total number of PCUs in 2030 were derived from future consumption levels of livestock products as predicted the Food and Agriculture Organization of the UN (*5*). The number of PCU in 2030 was projected in each country according to national growth rate. If national rates were not available the average continental growth rate was applied. Second, the proportions of animals that are raised in extensive and intensive production systems were projected based on Gilbert et al. (*6*) using forecasts of GDP per capita (PPP) from the *International Monetary Fund.* Forecast from 1980 to 2021 were extended to 2030 using linear regression models. Third we obtained the number of PCU in each production system (extensive vs intensive) in 2030, based on the future proportion of animals raised in each system.

Target 1. Imposing global regulations on antimicrobial use. Recent reports have shown that several high-income countries have highly productive livestock sectors while using less than 50 mg per PCU per year, and suggested this threshold as a ‘broadly reasonable target’ to limit antimicrobial consumption in the short term (*7*). First we estimate that by 2030 a cap on antimicrobial consumption at 50mg/PCU could be imposed in all countries currently exceeding this threshold (target 1A). Second, we consider the more realistic eventuality that these targets would only be enforced in the countries of the *Organisation for Economic Co-operation and Development* (OECD), as well as China (target 1B).

Target 2. Reduction in meat consumption. We estimated the total reduction in antimicrobial consumption that would be associated with a hypothetical decrease in meat consumption worldwide. First we consider that global meat consumption could be reduced to 40 g of meat/day (target 2A) as recently recommended in the revised Chinese nutritional guidelines (*8*). Second, we consider a more plausible target for reduction of meat consumption with the following conditions (target 2B):

- In countries with growing meat consumption per capita, the increase could be reduced twofold by 2030.
- In countries with decreasing meat consumption per capita, this decrease could be increased twofold by 2030.
- In all countries, total meat consumption should not exceed the median projected consumption in Europe in 2030.

We accounted for difference in productivity and population growth in each country; and calculated the ratio of PCU produced to the current levels of meat consumption. We scaled the number of PCU that would be produced under those consumption targets.

Target 3. User fee on veterinary antimicrobials. We followed a three-step procedure to evaluate the impact of imposing a user fee on antimicrobial use in animals. We estimated the shift in demand for veterinary antimicrobials, and the corresponding revenues associated with this policy. Five situations were considered to reflect a range of assumptions on fee rates and price elasticity of demand.

Step 1. Retail prices of veterinary antimicrobials.

Data on retail prices (P*) of products containing antimicrobials used in food animals were collected in 22 countries (Table S3), for 1,418 unique country-products combinations. Online searches for veterinary supply stores retailers were conducted in English, Mandarin Chinese, and Spanish using the following terms: 'veterinary supplies', 'antimicrobial purchase online', 'livestock antibiotics' and 'food animal antibiotics'. In addition, we obtained expert opinion estimates of prices of antimicrobials through contacts (phone and e-mail) with national branches of *veterinarians without borders*, as well as veterinarians in academic institutions. All price estimates were obtained between June and December 2016. All prices were expressed in US$ per kilogram of active ingredient.

In the 22 countries where information of retail prices of veterinary antimicrobial could be obtained, we calculated the median price for each class of antimicrobial in each county by aggregating the retail price of all products that contained a specific class of compound.

In the countries where information of retail prices of veterinary antimicrobial could not be obtained, the prices estimates were extrapolated from the 22 countries where data was available using the following procedure:

i) First, we classified countries in income groups (high-income *vs* low- and middle-income) based on a GNI per capita of 12,475 US$/year, as estimated by the World Bank for the fiscal year 2013.

ii) For each income group, we calculated the median price of each class of antimicrobial within that group. Median prices in high-income countries were calculated from 302 country-products samples, and median prices in low- and middle-income countries were based on 1,107 country-products samples.

iii) For the residual category 'other compounds', the price assigned was the median of the median price calculated for the 9 other classes of compounds.

We used this approach to extrapolate retail prices of antimicrobials worldwide in order to reflect potential variations in the relative prices of veterinary antimicrobials according to income. We used only two groups for the extrapolation of retail prices because of the limited number of countries were prices data could be obtained did not allow fine-scale regional extrapolations.

We performed an analysis of variance and multiple comparison test (Tukey) to study prices variations between classes and income groups (Fig. S3). This includes all classes that were represented by a least 10 price estimates (this excludes polymyxins and pleuromutilins). Prices of veterinary antimicrobial showed considerable variation within

each class of compounds and income groups. For the categories of antimicrobials where at least 10 estimates could be collected, significant prices differences were observed between those classes in high-income (p-value = .01) but not in low- and middle-income countries (p-value = .09). In high-income countries cephalosporins were significantly more expensive than penicillin, tetracycline and sulfonamides.

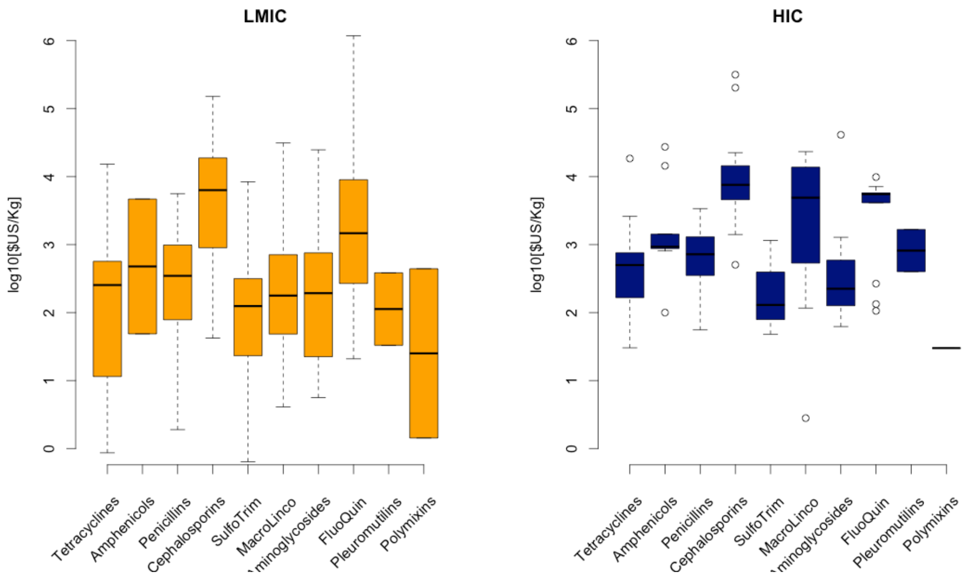

Fig. S3. Retail prices of one kilogram of active ingredient for 10 classes of veterinary antimicrobial in low- and middle-income countries (left) and high-income countries (right).

Step 2. Elasticity of Demand of Veterinary Antimicrobials.

Price elasticity of demand (PED) is percentage change in quantity of veterinary antimicrobials demanded (Q) in response to a change in price (P); in this analysis, this is after imposing a user fee on current prices.

$$PED = \frac{dQ/Q}{dP/P} \quad \text{(Eq. 1)}$$

Country-specific estimates of PED were calculated to estimate the global shift in demand for veterinary antimicrobials under a user fee imposed on current retail prices. Import data on the volume and sales value of tetracycline derivatives were obtained from the UN COMTRADE database (commodity code 294130). We used a log-log linear regression model (Eq 1) between yearly prices and imports volumes to estimate the PED (Fig S5).

$$\log P = \alpha \cdot logQ + logC \quad \text{(Eq.2)}$$

Where C is a constant and α is the regression coefficient to be estimated, such as: $PED = \frac{1}{\alpha}$. In each country, if a statistically significant estimate could not be obtain for α, than the median PED of the corresponding income group was considered for that country.

Overall, we obtained statistically significant estimates of PED for 67 countries that had 8 years (or more) of imports reported (Fig S4).

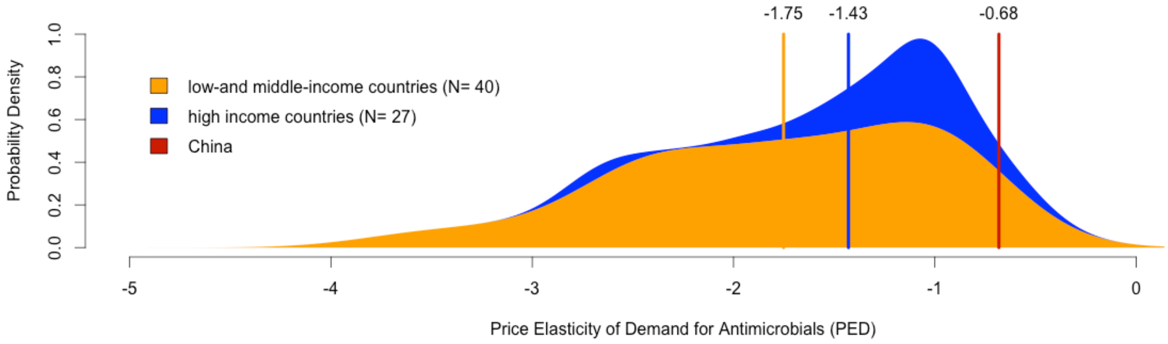

Fig. S4. Probability density of elasticity of demand (PED) by income groups.

We performed an analysis of variance (weighted by total antimicrobial consumption) – and excluding China- to identified a significant difference in elasticity (p-value < 0.05) between high-income countries and low-income countries. In low- and middle-income countries, the average demand was more elastic (PED = -1.75) than in high-income countries (PED = -1.43) with the notable exception of China (PED = -0.68).

Step 3. Shift in demand and associated revenues.

Taking the natural logarithm of Eq. 1 we obtain the relationship between price (P) and quantity (Q). At equilibrium (*) this can be rewritten:

$$P^* = {Q^*}^{\alpha} \cdot \exp(C) \quad \text{(Eq. 3)}$$

Applying an ad-valorem user fee of *TR* percent on the current prices of antimicrobials correspond to shift of in the demand curve (Fig. 4) given by:

$$P_t = Q_t^{\alpha} \cdot \exp(C) - TR \cdot P^* \text{ (Eq. 4)}$$

Where P_t and Q_t are the new price and quantity of antimicrobial demanded following the imposition of the user fee on the original price P*. In first approximation, we assumed a constant returns to scale in manufacturing, resulting in a horizontal supply curve such that, $P^* = P_t$. Combining Eq 2, 3 & 4, the quantity of antimicrobial demanded after imposing a user fee was given by:

$$Q_t = Q^* \cdot (1 + TR)^{PED} \quad \text{(Eq. 5)}$$

The revenues associated with the user fee in each country was given by quantity demanded Q_t times the different between the price paid by the consumer $P_c = (1 + TR) \cdot P^*$ -including the fee- and the original price $P_t = P^*$.

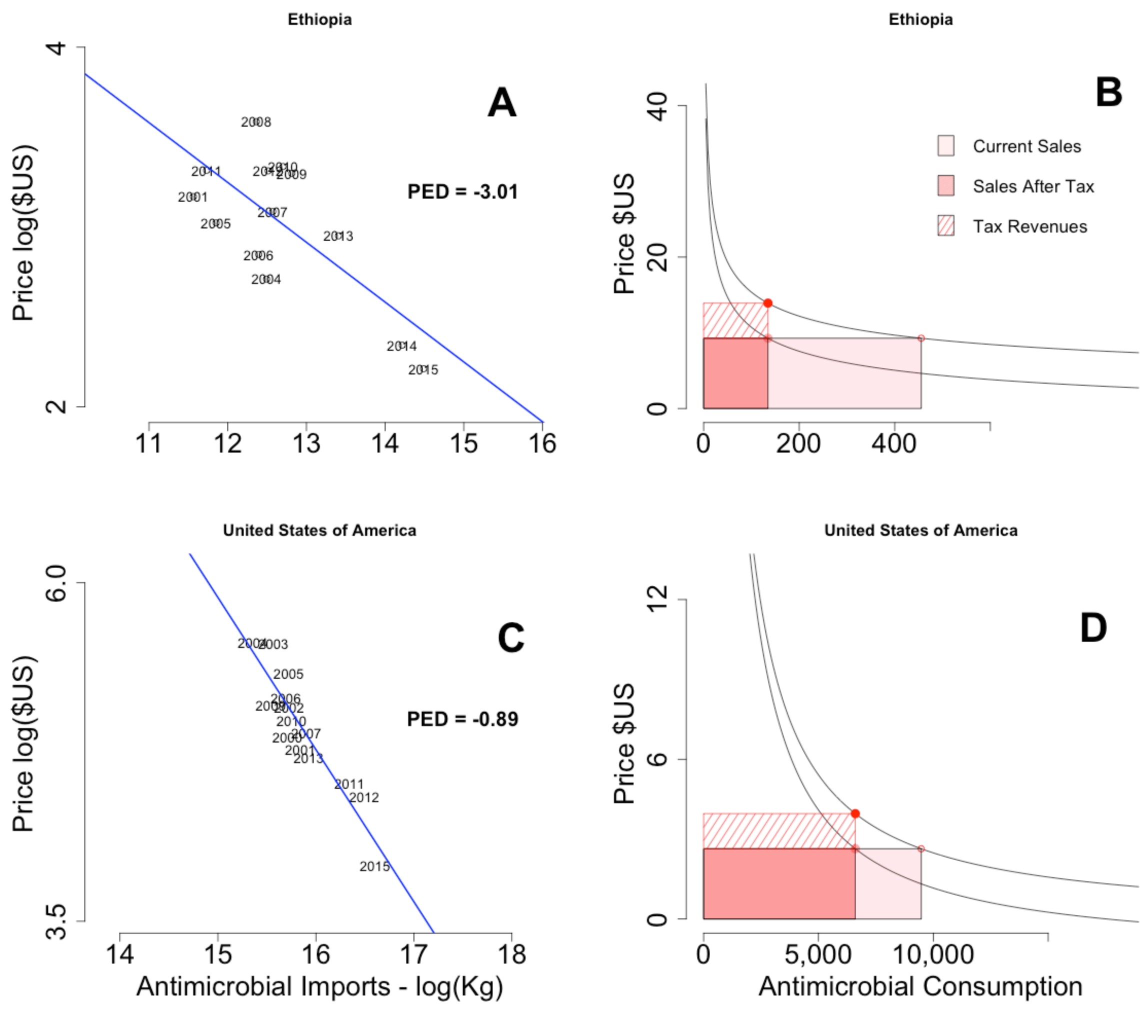

Fig. S5. Regression models for estimation of elasticity of demand (PED) in Ethiopia (A), and in the United States (C). Shift in demand curve and associated revenues from imposing a 50% user fee on veterinary antimicrobials in Ethiopia (B) and the United States (D).

In this study, the retail prices collected P* were scaled such as the total sales of antimicrobial would correspond to the reported size of the global market for veterinary antimicrobials. This price-scaling procedure maintains relative differences in prices across countries and compounds. The global animal health market, has been evaluated at 30 billion $US (HealthforAnimals.org, 2015). In 2007, the global market for anti-infective and medicated feed additives was estimate at 27% of the animal health market (*9*). However, in the absence of public information on the current share of antimicrobials in the animal health market, we hypothesize that antimicrobials could represent between 15% (4.5 billions) to 40% (12 billions) of the current market. This generates a lower and upper bound on the estimates of revenues associated with a user fee policy for this study. The reference scenario (3C) used for the user fee policy assumed a rate of 50% and PED estimates derived from imports. In addition we conducted a sensitivity analysis on the

rate of the user fee (scenario 3D and 3E) as well as on the estimates of PED derived from import data (scenario 3A and 3B).

Combined interventions (Targets 1, 2 & 3).

The three intervention policies were also considered in combination. In the absence of information on potential synergetic (or antagonist effect) between the scenario we assumed that the effect interventions strategies to be additive if applied sequentially. The hypothetical reduction in meat consumption was applied first (Scenario 2B), followed by either imposing a user fee directly (Scenario 2B+3C), or imposing a cap on consumption at 50mg/PCU in OECD countries and China (Scenario 2B+1B). We also considered all three interventions together (Scenario 2B+1B+3C). In this case, meat consumption was decreased first, and the user fee was applied on the residual consumption of antimicrobials after imposing the regulation cap.

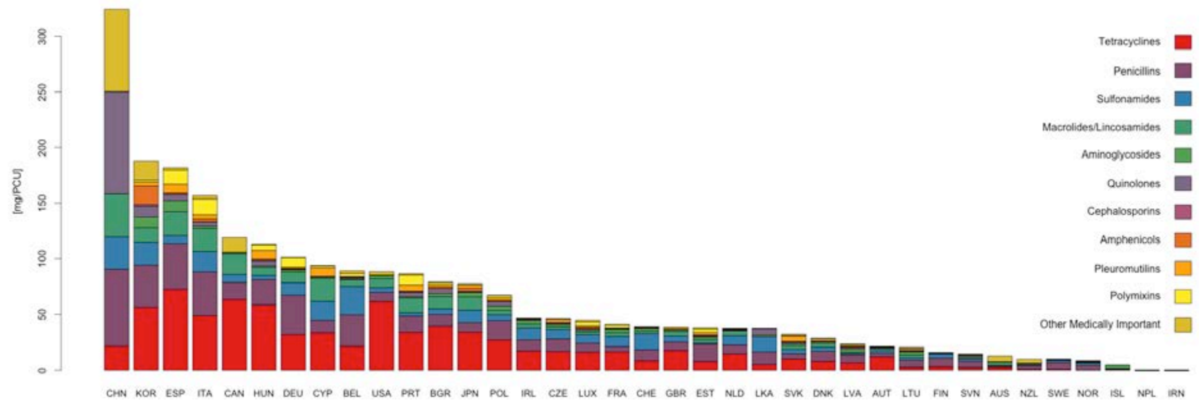

Fig S1. Reported sales of veterinary antimicrobials. Consumption levels in milligrams per population correction unit for 10 classes of compounds (Table S1).

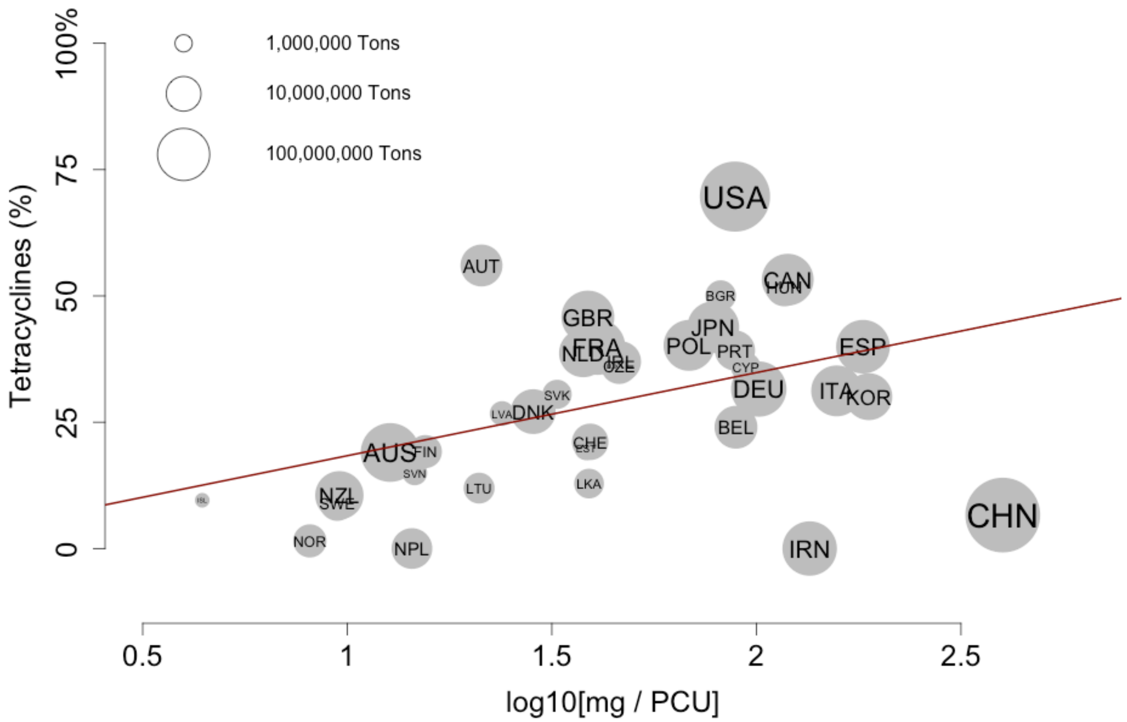

Fig. S2. Share of tetracycline in a country vs antimicrobial consumption per kilogram of animal. Circles are proportional to the size of the livestock sector within each country.

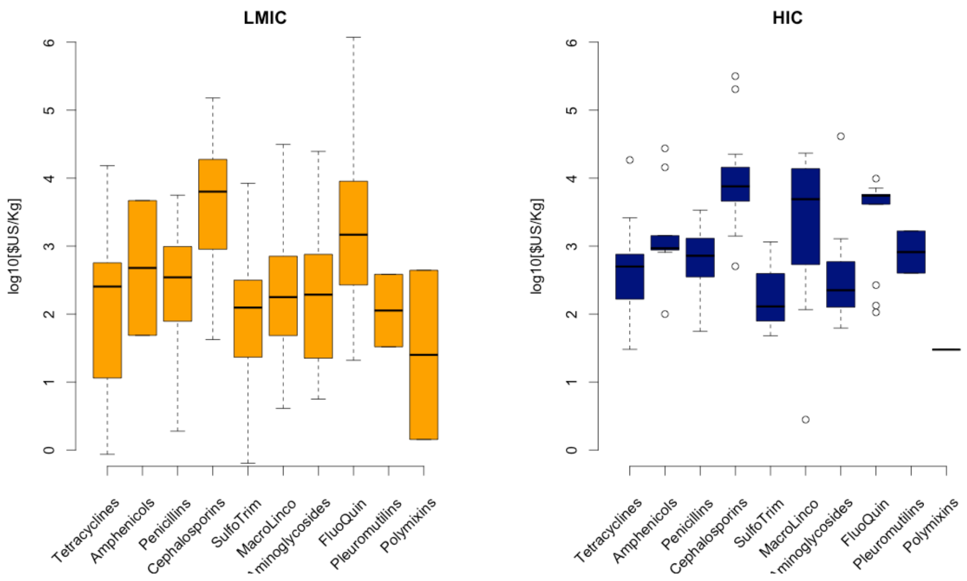

Fig. S3. Retail prices of one kilogram of active ingredient for 10 classes of veterinary antimicrobial in low- and middle-income countries (left) and high-income countries (right).

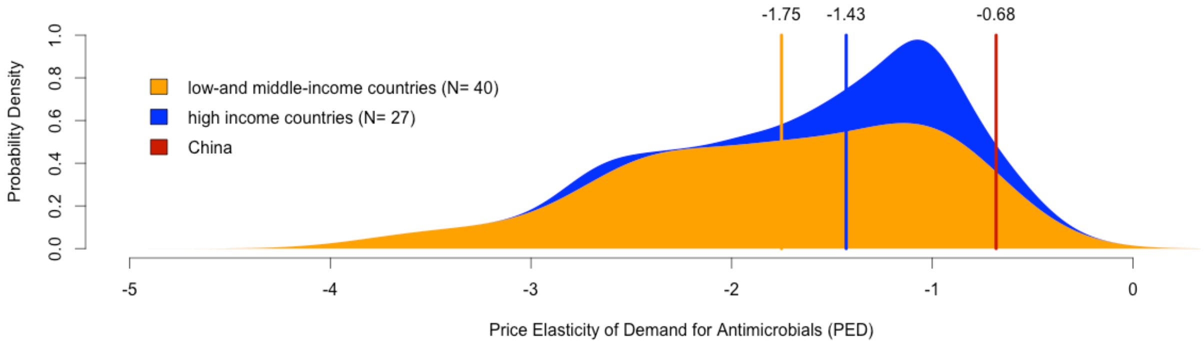

Fig. S4. Probability density of elasticity of demand (PED) by income groups..

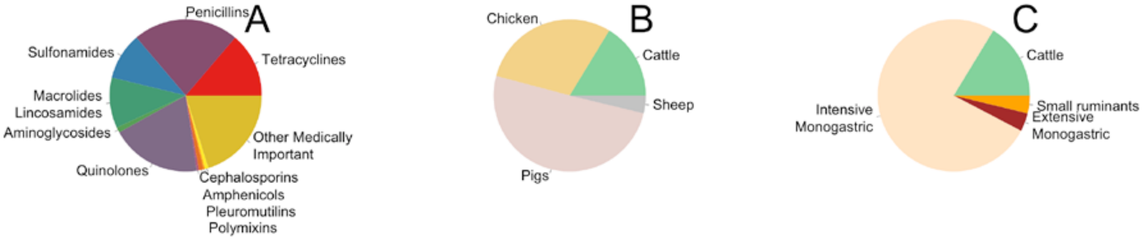

Fig. S6. Global antimicrobial consumption by class of drug (A), species (B), and class of animal (C).

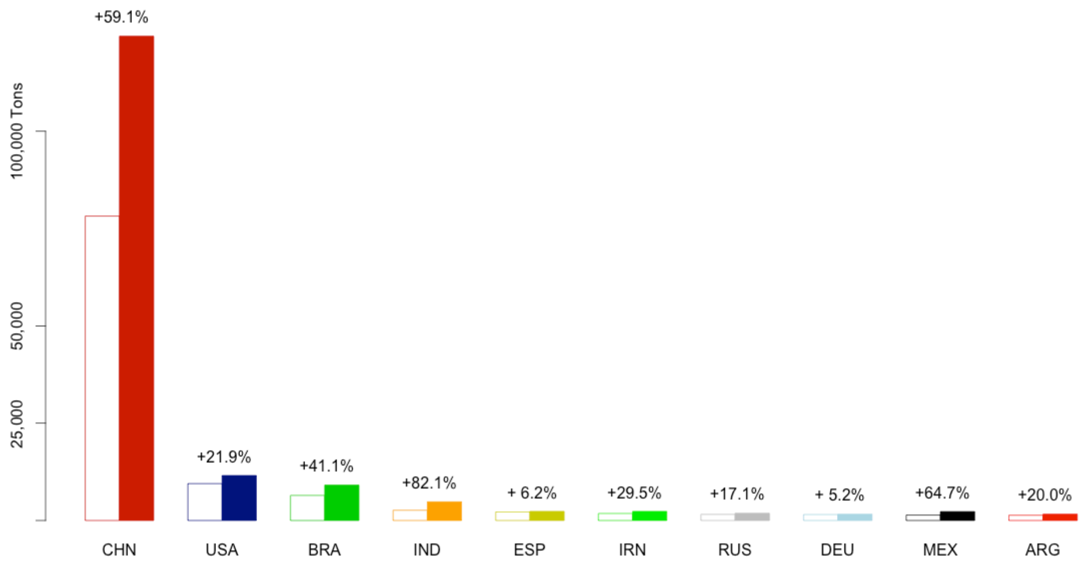

Fig. S7. Antimicrobial consumption in largest consumers in 2013 and 2030.

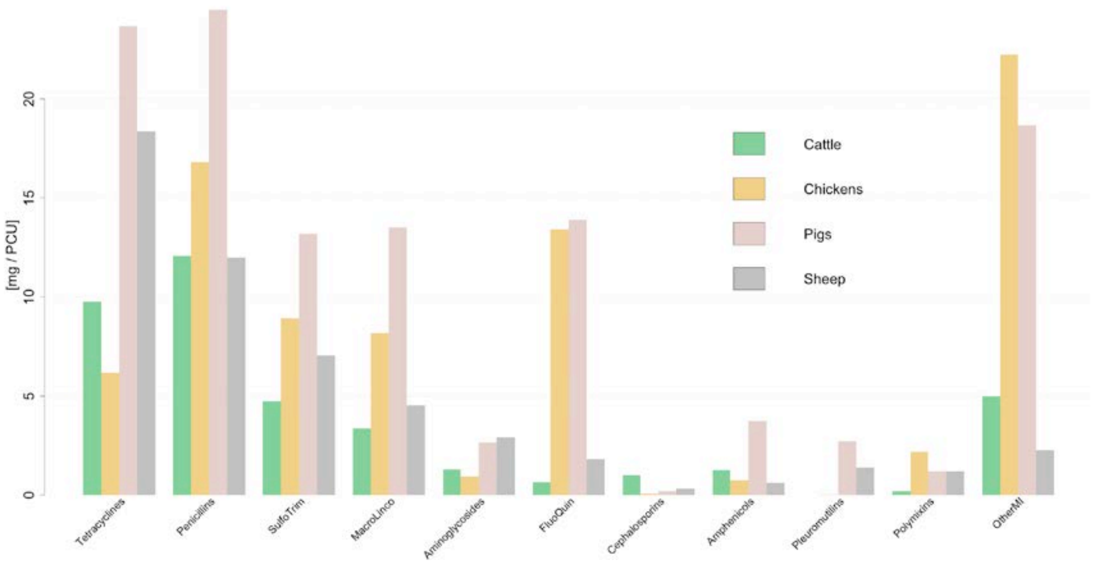

Fig. S8. Antimicrobial consumption per population correction unit (PCU) by class of antimicrobials.

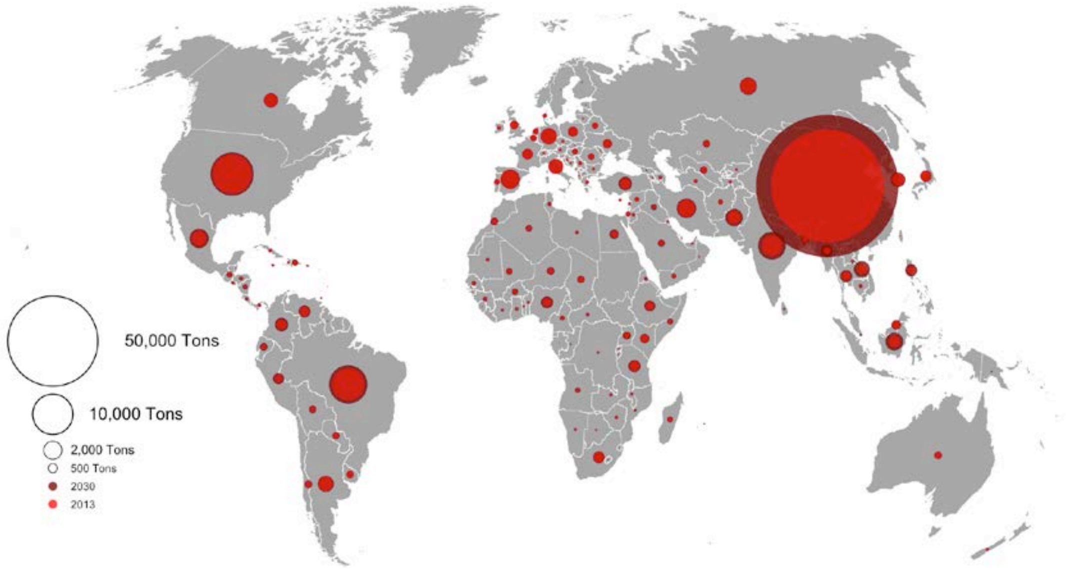

Fig. S9. Antimicrobial consumption for food animal production by country, in 2013 (light red) and projected for 2030 (dark red).

Table S1. Reported antimicrobial sales in (Kg)

Country	Source	Year	Animal	Tetracyclines	Penicillins	Sulfonamides	Macrolides	Aminoglycosides	Quinolones	Cephalosporins	Amphenicols	Pleuromutilins	Polymixins	Other	Total Medicaly Important
Australia	Department of Agriculture and Department of Health	2010	Cattle	NA	NA	NA	NA	NA	NA	NA	NA	NA	NA	NA	22510
Australia	Department of Agriculture and Department of Health	2010	Sheep	NA	NA	NA	NA	NA	NA	NA	NA	NA	NA	NA	26990
Australia	Department of Agriculture and Department of Health	2010	Chicken	NA	NA	NA	NA	NA	NA	NA	NA	NA	NA	NA	163100
Australia	Department of Agriculture and Department of Health	2010	Pig	NA	NA	NA	NA	NA	NA	NA	NA	NA	NA	NA	104200
Australia	Department of Agriculture and Department of Health	2010	Total	58000	26320	13300	71330	5620	0	300	900	NA	NA	126840	305050
Austria	European Medicine Agency	2011	Pig	NA	NA	NA	737.2	NA	38	41.8	NA	NA	NA	12840.2	13657.2
Austria	European Medicine Agency	2013	Total	30700	8600	6100	5000	1300	600	400	300	400	900	500	54800
Belgium	European Medicine Agency / BelVetSAC	2009	Cattle	NA	NA	NA	NA	NA	NA	NA	NA	NA	NA	NA	30977.73567
Belgium	European Medicine Agency / BelVetSAC	2009	Chicken	NA	NA	NA	NA	NA	NA	NA	NA	NA	NA	NA	32575.79346
Belgium	European Medicine Agency / BelVetSAC	2009	Pig	NA	NA	NA	NA	NA	NA	NA	NA	NA	NA	NA	195946.4709
Belgium	European Medicine Agency / BelVetSAC	2013	Total	62300	82200	74500	17300	1300	3200	900	1500	1600	7800	6900	259500
Bulgaria	European Medicine Agency	2013	Total	23300	6100	3000	6400	1500	2800	30	900	1100	1100	300	46530
China	Zhang et al 2015	2013	Pig	3300000	10600000	3520000	5840000	NA	13900000	NA	NA	NA	NA	11300000	48400000
China	Zhang et al 2015	2013	Chicken	1130000	3630000	2450000	2070000	NA	4870000	NA	NA	NA	NA	3970000	18100000
China	Zhang et al 2015	2013	Total	5180000	16710000	6985000	9300000	NA	22200000	NA	NA	NA	NA	17890000	78200000
Cyprus	European Medicine Agency	2013	Total	17200	5500	8800	10500	600	130	104	100	3900	900	200	47934
Canada	CIPARS	2014	Total	599540	146391	67206	172204	13273	370	2523	NA	NA	NA	125139	1126646
Czech Republic	European Medicine Agency	2013	Total	20700	13900	10700	3000	2400	1230	500	300	3300	800	400	57230
Denmark	European Medicine Agency / DANMAP	2014	Pig	26850	24459	8074	13116	4494	4	4	244	8120	NA	655	86020
Denmark	European Medicine Agency / DANMAP	2014	Chicken	618	446	83	369	21	0	0	9	0	0	3	1549
Denmark	European Medicine Agency / DANMAP	2014	Total	29216	34801	13402	13684	5014	1688	109	1089	8120	NA	680	107803
Estonia	European Medicine Agency	2013	Total	1700	3500	250	700	500	200	200	40	500	700	300	8590
Finland	Thompson et al	2013	Chicken	NA	NA	NA	NA	NA	NA	NA	NA	NA	NA	NA	36.5
Finland	European Medicine Agency	2013	Total	2400	6200	3000	600	30	100	40	100	40	0	0	12510
France	ANSES	2013	Cattle	NA	NA	NA	NA	NA	NA	NA	NA	NA	NA	NA	147170
France	ANSES	2013	Ho	NA	NA	NA	NA	NA	NA	NA	NA	NA	NA	NA	15170
France	ANSES	2013	Ra	NA	NA	NA	NA	NA	NA	NA	NA	NA	NA	NA	52460
France	ANSES	2013	Sheep	NA	NA	NA	NA	NA	NA	NA	NA	NA	NA	NA	45160
France	ANSES	2013	Pig	NA	NA	NA	NA	NA	NA	NA	NA	NA	NA	NA	262120
France	ANSES	2013	Chicken	NA	NA	NA	NA	NA	NA	NA	NA	NA	NA	NA	156570
France	European Medicine Agency	2013	Total	271800	81000	152800	55300	51900	9100	3800	4700	5600	42400	2500	680900
Germany	Merle et al 2012	2011	Chicken	261	25147	4305	20873	2179	823	0	NA	NA	42648	4664	100900
Germany	Merle et al 2013	2011	Pig	267122	246756	103787	80844	9113	958	397	866	6664	31516	226477	974500
Germany	Merle et al 2014	2011	Cattle	22075	52997	25178	2559	3793	3539	3394	1740	0	2168	109357	226800
Germany	European Medicine Agency	2013	Total	482000	531600	171600	143400	28600	11700	4100	5100	18800	124700	5500	1527100
Hungary	European Medicine Agency	2013	Total	90700	35000	6000	11400	1900	7200	300	1800	12200	7600	1400	175500
Iceland	European Medicine Agency	2013	Total	30	30	50	0	200	4	0	0	0	0	0	314
Iran	Aalipour et al 2014	2010	Chicken	NA	NA	NA	NA	NA	NA	NA	NA	NA	NA	NA	607206
Iran	Aalipour et al 2015	2010	Cattle	NA	NA	NA	NA	NA	NA	NA	NA	NA	NA	NA	330669
Iran	Aalipour et al 2016	2010	Sheep	NA	NA	NA	NA	NA	NA	NA	NA	NA	NA	NA	869020
Iran	Aalipour et al 2017	2010	Total	NA	NA	NA	NA	NA	NA	NA	NA	NA	NA	NA	1806896
Ireland	European Medicine Agency	2013	Total	36900	20900	23700	6700	7100	900	900	1900	10	100	600	99710
Italy	European Medicine Agency	2013	Total	411500	330900	153700	174200	17900	31500	2300	18000	32200	120600	25800	1318600
Latvia	European Medicine Agency	2013	Total	1700	2000	340	320	800	400	140	20	300	300	40	6360
Lithuania	European Medicine Agency	2013	Total	1500	3800	1300	1700	1700	500	200	200	1100	40	500	12540
Luxembourg	European Medicine Agency	2013	Total	1000	500	480	140	100	80	40	100	30	200	100	2770
Japan	Ministry of Agriculture, Forestry and Fisheries	2014	Cattle	17857.8	17398.3	9142.6	1243.1	3267.7	929.2	2687.9	1923.9	0	0	162.1	54612
Japan	Ministry of Agriculture, Forestry and Fisheries	2014	Chicken	25218.8	16703.6	20974.1	14725.1	10368.9	2470.7	0	1670.4	0	0	0	92131
Japan	Ministry of Agriculture, Forestry and Fisheries	2014	Pig	232885.7	30697.6	70935.3	71541.4	24457	1530.4	372.7	19047.6	28241.6	9971.1	28.9	489709
Japan	Ministry of Agriculture, Forestry and Fisheries	2014	Total	324965	79529.2	110549.5	111219.7	38662.3	6581.5	3060.6	26136	28241.6	9971.1	1734.8	741779
South Korea	Animal and Plant Quarantine Agency	2012	Cattle	8784	33336	3709	2225	6678	1122	2660	3300	0	640	489	62943
South Korea	Animal and Plant Quarantine Agency	2012	Pig	77241	120169	70052	45378	27341	5959	4055	65751	17343	6060	10274	449623
South Korea	Animal and Plant Quarantine Agency	2012	Chicken	24053	16471	19459	5382	6183	35394	1144	12267	397	2209	26491	149450
South Korea	Animal and Plant Quarantine Agency	2012	Total	281974	189748	102340	65096	46071	49500	7873	83423	17740	8909	85490	938164
Nepal	Global Antimicrobial Resistance Partnership	2012	Total	NA	NA	NA	NA	NA	NA	NA	NA	NA	NA	NA	31595
Netherlands	MARAN / Wageningen	2014	Cattle	36945	12741	14411	17099	457	1744	19.47	3697	0	168	1080	88361
Netherlands	MARAN / Wageningen	2014	Pig	35679	15680	19331	7692	44	486.19	0	907	704	1079	780	82382
Netherlands	MARAN / Wageningen	2014	Chicken	2427	10746	3116	1463	96	1147	0	0	0	10.32	306	19311
Netherlands	European Medicine Agency	2013	Total	87200	47400	50400	25700	3600	2800	220	3600	1400	2000	1100	225420
New Zealand	Ministry for Primary Industries	2011	Cattle	0	7000	0	0	0	0	1000	0	0	0	0	8000
New Zealand	Ministry for Primary Industries	2011	Sheep	NA	NA	NA	NA	NA	NA	NA	NA	NA	NA	NA	2500
New Zealand	Ministry for Primary Industries	2011	Total	6019.37	15682.54	4696.43	6524.07	1234.95	39.67	1707.3	NA	NA	NA	21138.4	57100.13
Norway	European Medicine Agency	2013	Total	100	3000	1800	3	600	710	1	300	100	0	0	6614
Poland	European Medicine Agency	2013	Total	231400	147400	47500	34800	32600	33900	1800	6500	19800	16800	3000	575500
Portugal	European Medicine Agency	2013	Total	70300	30300	6100	27300	2300	8100	1100	1300	11700	18200	2600	179300
Slovakia	European Medicine Agency	2013	Total	4800	2400	1900	1500	800	730	200	200	2300	300	600	15730
Slovenia	European Medicine Agency	2013	Total	600	1600	600	200	400	303	40	100	50	10	100	4003
Spain	European Medicine Agency	2013	Total	879600	498600	85400	259400	121000	68700	2400	14300	95600	149000	28000	2202000
Sri Lanka		2012	Total	2590	6206	7274	0	808	3256	8	0	0	0	0	20142
Sweden	European Medicine Agency	2014	Pig	382.1	1399.3	543.8	293	53.9	3.2	0	2.3	112.5	79.7	NA	2869.8
Sweden	European Medicine Agency	2013	Total	900	6100	2000	400	300	40	4	100	100	100	0	10044
Switzerland	ARCH-Vet	2014	Total	10402	12643	18111	2858	3125	404	522	188	NA	773	222	49248
United Kingdom	Veterinary Medicine Directorate	2013	Cattle	NA	NA	NA	NA	NA	NA	NA	NA	NA	NA	NA	14000
United Kingdom	Veterinary Medicine Directorate	2013	Pig	NA	NA	NA	NA	NA	NA	NA	NA	NA	NA	NA	91163
United Kingdom	Veterinary Medicine Directorate	2013	Chicken	NA	NA	NA	NA	NA	NA	NA	NA	NA	NA	NA	134836
United Kingdom	European Medicine Agency	2013	Total	193300	81000	59100	45900	14800	2400	1800	NA	11400	NA	12200	421900
United States	Food and Drug Administration	2014	Total	6600849	885975	452224	855450	304160	17220	31722	NA	NA	NA	328389	9475989
Vietnam	Carrique-Mas et al 2014	2013	Chicken	NA	43846	11701	11916	NA	10962	0	NA	NA	NA	134156	212581

Individual reports for each country are accessible at (https://www.dropbox.com/sh/fsht4fp0qsquf2u/AADCArtJJCk2ovrKPh_wHEbga?dl=0)

Table S2. Projected tons of antimicrobials by country 2013 (+%Growth by 2030)

Country	Total	Tetracyclines	Penicillins	Sulfonamides	Macrolides	Aminoglycosides	Quinolones	Cephalosporins	Amphenicols	Pleuromutilins	Polymixins	Others
Afghanistan	112 (+49%)	32 (+%)	31 (+%)	14 (+%)	10 (+%)	5 (+%)	3 (+%)	2 (+%)	3 (+%)	1 (+%)	1 (+%)	11 (+%)
Albania	18 (+3%)	4 (+0%)	4 (+0%)	2 (+0%)	2 (+0%)	1 (+0%)	1 (+0%)	0 (%)	0 (%)	0 (%)	0 (%)	3 (+0%)
Algeria	167 (+71%)	29 (+52%)	42 (+71%)	22 (+68%)	16 (+75%)	6 (+50%)	15 (+87%)	1 (+100%)	3 (+33%)	2 (+50%)	4 (+75%)	27 (+89%)
American Samoa	1 (+-88%)	0 (+%)	0 (+%)	0 (+%)	0 (+%)	0 (+%)	0 (+%)	0 (+%)	0 (+%)	0 (+%)	0 (+%)	0 (+%)
Andorra	0 (%)	0 (+%)	0 (+%)	0 (+%)	0 (+%)	0 (+%)	0 (+%)	0 (+%)	0 (+%)	0 (+%)	0 (+%)	0 (+%)
Angola	92 (+106%)	24 (+104%)	24 (+104%)	11 (+100%)	8 (+112%)	3 (+100%)	5 (+120%)	1 (+200%)	2 (+150%)	1 (+100%)	1 (+100%)	12 (+117%)
Anguilla	0 (%)	0 (+%)	0 (+%)	0 (+%)	0 (+%)	0 (+%)	0 (+%)	0 (+%)	0 (+%)	0 (+%)	0 (+%)	0 (+%)
Antigua and Barbuda	0 (%)	0 (%)	0 (%)	0 (%)	0 (%)	0 (%)	0 (%)	0 (%)	0 (%)	0 (%)	0 (%)	0 (%)
Argenti	1349 (+20%)	313 (+15%)	351 (+18%)	152 (+20%)	120 (+21%)	35 (+14%)	86 (+34%)	21 (+14%)	33 (+15%)	3 (+0%)	16 (+25%)	220 (+25%)
Armenia	13 (+61%)	3 (+67%)	3 (+67%)	1 (+100%)	1 (+100%)	0 (%)	1 (+0%)	0 (%)	0 (%)	0 (%)	0 (%)	2 (+50%)
Aruba	0 (%)	0 (+%)	0 (+%)	0 (+%)	0 (+%)	0 (+%)	0 (+%)	0 (+%)	0 (+%)	0 (+%)	0 (+%)	0 (+%)
Australia	305 (+19%)	60 (+13%)	77 (+19%)	36 (+19%)	34 (+21%)	9 (+11%)	20 (+25%)	1 (+100%)	6 (+17%)	4 (+0%)	12 (+25%)	46 (+24%)
Austria	55 (+3%)	9 (+0%)	13 (+0%)	6 (+0%)	5 (+0%)	1 (+0%)	3 (+0%)	1 (+0%)	1 (+0%)	0 (%)	1 (+0%)	16 (+6%)
Azerbaijan	53 (+68%)	6 (+50%)	15 (+60%)	7 (+57%)	5 (+80%)	1 (+100%)	5 (+80%)	1 (+0%)	1 (+100%)	0 (%)	1 (+100%)	11 (+73%)
Bahamas	2 (+73%)	0 (%)	0 (%)	0 (%)	0 (%)	0 (%)	0 (%)	0 (%)	0 (%)	0 (%)	0 (%)	1 (+0%)
Bahrain	3 (+41%)	1 (+%)	1 (+%)	0 (+%)	0 (+%)	0 (+%)	0 (+%)	0 (+%)	0 (+%)	0 (+%)	0 (+%)	0 (+%)
Bangladesh	202 (+69%)	52 (+%)	55 (+%)	25 (+%)	18 (+%)	8 (+%)	8 (+%)	3 (+%)	5 (+%)	2 (+%)	3 (+%)	24 (+%)
Barbados	4 (+86%)	0 (%)	1 (+100%)	0 (%)	0 (%)	0 (%)	1 (+0%)	0 (%)	0 (%)	0 (%)	0 (%)	1 (+100%)
Belarus	223 (+10%)	47 (+6%)	50 (+10%)	24 (+12%)	22 (+9%)	5 (+0%)	23 (+13%)	2 (+0%)	5 (+20%)	2 (+0%)	3 (+33%)	41 (+10%)
Belgium	260 (+6%)	57 (+%)	59 (+%)	32 (+%)	28 (+%)	6 (+%)	12 (+%)	0 (+%)	6 (+%)	7 (+%)	5 (+%)	46 (+%)
Belize	6 (+58%)	1 (+0%)	1 (+100%)	1 (+0%)	1 (+0%)	0 (%)	1 (+0%)	0 (%)	0 (%)	0 (%)	0 (%)	1 (+100%)
Benin	36 (+69%)	9 (+56%)	10 (+70%)	4 (+75%)	3 (+67%)	1 (+100%)	1 (+100%)	1 (+0%)	1 (+100%)	0 (%)	0 (%)	5 (+80%)
Bermuda	0 (%)	0 (+%)	0 (+%)	0 (+%)	0 (+%)	0 (+%)	0 (+%)	0 (+%)	0 (+%)	0 (+%)	0 (+%)	0 (+%)
Bhutan	4 (+71%)	1 (+100%)	1 (+100%)	1 (+0%)	0 (%)	0 (%)	0 (%)	0 (%)	0 (%)	0 (%)	0 (%)	1 (+0%)
Bolivia (Pluritiol State of)	187 (+92%)	30 (+67%)	44 (+89%)	22 (+86%)	18 (+94%)	4 (+75%)	21 (+114%)	1 (+100%)	3 (+100%)	1 (+0%)	4 (+75%)	39 (+110%)
Bosnia and Herzegovi	26 (+14%)	5 (+0%)	6 (+17%)	3 (+0%)	3 (+0%)	1 (+0%)	3 (+0%)	0 (%)	1 (+0%)	0 (%)	0 (%)	5 (+20%)
Botswa	27 (+41%)	6 (+33%)	8 (+50%)	3 (+67%)	2 (+100%)	1 (+0%)	1 (+0%)	1 (+0%)	1 (+0%)	0 (%)	0 (%)	4 (+25%)
Brazil	6448 (+41%)	1342 (+39%)	1572 (+39%)	715 (+41%)	590 (+43%)	142 (+37%)	566 (+48%)	77 (+29%)	136 (+41%)	16 (+88%)	94 (+44%)	1199 (+42%)
British Virgin Islands	0 (%)	0 (+%)	0 (+%)	0 (+%)	0 (+%)	0 (+%)	0 (+%)	0 (+%)	0 (+%)	0 (+%)	0 (+%)	0 (+%)
Brunei Darussalam	10 (+110%)	0 (%)	2 (+150%)	1 (+100%)	1 (+100%)	0 (%)	2 (+100%)	0 (%)	0 (%)	0 (%)	0 (%)	3 (+100%)
Bulgaria	47 (+8%)	10 (+0%)	10 (+10%)	5 (+20%)	5 (+0%)	1 (+0%)	5 (+20%)	0 (%)	1 (+0%)	0 (%)	1 (+0%)	9 (+11%)
Burki Faso	117 (+115%)	32 (+106%)	33 (+112%)	14 (+114%)	10 (+120%)	4 (+125%)	3 (+167%)	2 (+150%)	3 (+133%)	1 (+0%)	1 (+200%)	13 (+131%)
Burundi	13 (+89%)	3 (+133%)	3 (+133%)	2 (+50%)	1 (+100%)	0 (%)	0 (%)	0 (%)	0 (%)	0 (%)	0 (%)	1 (+200%)
Cote d'Ivoire	43 (+60%)	9 (+56%)	12 (+58%)	5 (+60%)	4 (+50%)	1 (+100%)	3 (+33%)	1 (+0%)	1 (+100%)	0 (%)	1 (+0%)	7 (+57%)
Cabo Verde	1 (+140%)	0 (%)	0 (%)	0 (%)	0 (%)	0 (%)	0 (%)	0 (%)	0 (%)	0 (%)	0 (%)	0 (%)
Cambodia	55 (+97%)	14 (+%)	15 (+%)	6 (+%)	5 (+%)	2 (+%)	2 (+%)	1 (+%)	2 (+%)	0 (+%)	0 (+%)	8 (+%)
Cameroon	94 (+58%)	24 (+54%)	26 (+54%)	11 (+64%)	8 (+62%)	3 (+67%)	4 (+75%)	2 (+50%)	2 (+100%)	0 (%)	1 (+100%)	12 (+75%)
Cada	1127 (+14%)	256 (+12%)	233 (+13%)	118 (+14%)	111 (+14%)	21 (+14%)	126 (+15%)	5 (+20%)	25 (+12%)	13 (+8%)	16 (+19%)	203 (+15%)
Cayman Islands	0 (%)	0 (+%)	0 (+%)	0 (+%)	0 (+%)	0 (+%)	0 (+%)	0 (+%)	0 (+%)	0 (+%)	0 (+%)	0 (+%)
Central African Republic	62 (+51%)	17 (+53%)	17 (+53%)	8 (+50%)	5 (+60%)	2 (+100%)	2 (+0%)	1 (+100%)	2 (+0%)	0 (%)	1 (+0%)	7 (+43%)
Chad	201 (+76%)	49 (+78%)	61 (+75%)	25 (+76%)	18 (+72%)	7 (+86%)	4 (+75%)	5 (+60%)	6 (+83%)	1 (+0%)	1 (+200%)	24 (+79%)
Chile	270 (+39%)	50 (+36%)	58 (+38%)	30 (+37%)	27 (+41%)	5 (+40%)	33 (+42%)	1 (+100%)	5 (+40%)	2 (+50%)	5 (+40%)	54 (+43%)
"Chi, Hong Kong SAR"	5 (+133%)	0 (+%)	1 (+%)	1 (+%)	0 (+%)	0 (+%)	1 (+%)	0 (+%)	0 (+%)	0 (+%)	0 (+%)	1 (+%)
"Chi, Macao SAR"	1 (+-100%)	0 (+%)	0 (+%)	0 (+%)	0 (+%)	0 (+%)	0 (+%)	0 (+%)	0 (+%)	0 (+%)	0 (+%)	0 (+%)
"Chi, mainland"	78200 (+59%)	6758 (+56%)	16815 (+58%)	7075 (+67%)	9069 (+59%)	153 (+29%)	20820 (+59%)	77 (+57%)	100 (+54%)	30 (+-23%)	40 (+8%)	17264 (+59%)
"Chi, Taiwan Province of"	3 (+62%)	1 (+%)	1 (+%)	0 (+%)	0 (+%)	0 (+%)	0 (+%)	0 (+%)	0 (+%)	0 (+%)	0 (+%)	0 (+%)
Colombia	630 (+83%)	126 (+63%)	161 (+75%)	72 (+81%)	58 (+90%)	15 (+60%)	51 (+124%)	9 (+33%)	14 (+71%)	1 (+200%)	9 (+100%)	115 (+99%)
Comoros	1 (+41%)	0 (+%)	0 (+%)	0 (+%)	0 (+%)	0 (+%)	0 (+%)	0 (+%)	0 (+%)	0 (+%)	0 (+%)	0 (+%)
Congo	7 (+114%)	2 (+50%)	2 (+100%)	1 (+100%)	1 (+0%)	0 (%)	0 (%)	0 (%)	0 (%)	0 (%)	0 (%)	1 (+100%)
Cook Islands	0 (%)	0 (+%)	0 (+%)	0 (+%)	0 (+%)	0 (+%)	0 (+%)	0 (+%)	0 (+%)	0 (+%)	0 (+%)	0 (+%)
Costa Rica	56 (+61%)	10 (+40%)	13 (+54%)	6 (+67%)	5 (+80%)	1 (+100%)	6 (+83%)	0 (%)	1 (+100%)	0 (%)	1 (+100%)	11 (+73%)
Croatia	35 (+8%)	7 (+14%)	8 (+0%)	4 (+0%)	4 (+0%)	1 (+0%)	4 (+0%)	0 (%)	1 (+0%)	1 (+0%)	0 (%)	6 (+0%)
Cuba	77 (+14%)	1 (+%)	29 (+%)	12 (+%)	9 (+%)	3 (+%)	4 (+%)	2 (+%)	3 (+%)	0 (+%)	1 (+%)	14 (+%)
Cyprus	48 (+20%)	14 (+7%)	10 (+20%)	6 (+0%)	4 (+25%)	2 (+0%)	4 (+50%)	0 (%)	1 (+0%)	1 (+0%)	1 (+0%)	5 (+60%)
Czech Republic	57 (+7%)	11 (+0%)	13 (+0%)	6 (+17%)	6 (+0%)	1 (+0%)	7 (+0%)	0 (%)	1 (+0%)	1 (+0%)	1 (+0%)	11 (+9%)
Democratic People's Republic of Korea	5 (+%)	34 (+-29%)	8 (+%)	8 (+%)	4 (+%)	3 (+%)	1 (+%)	2 (+%)	0 (+%)	1 (+%)	0 (+%)	0 (+%)
Democratic Republic of the Congo	19 (+115%)	5 (+120%)	5 (+100%)	2 (+150%)	2 (+100%)	1 (+100%)	1 (+100%)	0 (%)	0 (%)	0 (%)	0 (%)	2 (+150%)

Denmark	108 (+5%)	32 (+%)	31 (+%)	11 (+%)	15 (+%)	5 (+%)	0 (+%)	1 (+%)	1 (+%)	8 (+%)	0 (+%)	3 (+%)
Djibouti	2 (+40%)	1 (+%)	0 (+%)	0 (+%)	0 (+%)	0 (+%)	0 (+%)	0 (+%)	0 (+%)	0 (+%)	0 (+%)	0 (+%)
Dominica	0 (%)	0 (%)	0 (%)	0 (%)	0 (%)	0 (%)	0 (%)	0 (%)	0 (%)	0 (%)	0 (%)	0 (%)
Dominican Republic	155 (+61%)	24 (+54%)	37 (+59%)	18 (+56%)	15 (+60%)	3 (+67%)	17 (+71%)	1 (+100%)	3 (+67%)	0 (%)	3 (+67%)	33 (+67%)
Ecuador	186 (+97%)	30 (+77%)	42 (+95%)	21 (+95%)	18 (+100%)	3 (+100%)	23 (+109%)	1 (+100%)	3 (+100%)	1 (+0%)	4 (+75%)	40 (+108%)
Egypt	277 (+98%)	24 (+58%)	72 (+92%)	35 (+97%)	29 (+100%)	7 (+71%)	33 (+124%)	3 (+33%)	5 (+60%)	1 (+100%)	6 (+117%)	62 (+115%)
El Salvador	37 (+94%)	5 (+80%)	9 (+89%)	4 (+100%)	4 (+75%)	1 (+0%)	4 (+125%)	0 (%)	1 (+0%)	0 (%)	1 (+0%)	8 (+112%)
Equatorial Guinea	0 (%)	0 (%)	0 (%)	0 (%)	0 (%)	0 (%)	0 (%)	0 (%)	0 (%)	0 (%)	0 (%)	0 (%)
Eritrea	50 (+91%)	13 (+%)	15 (+%)	6 (+%)	4 (+%)	2 (+%)	1 (+%)	1 (+%)	1 (+%)	0 (+%)	0 (+%)	6 (+%)
Estonia	9 (+1%)	2 (+0%)	2 (+0%)	1 (+0%)	1 (+0%)	0 (%)	1 (+0%)	0 (%)	0 (%)	0 (%)	0 (%)	2 (+0%)
Ethiopia	456 (+66%)	92 (+62%)	145 (+64%)	60 (+67%)	42 (+69%)	18 (+61%)	10 (+100%)	11 (+64%)	14 (+64%)	2 (+50%)	4 (+75%)	57 (+72%)
Falkland Islands (Malvis)	1 (+-42%)	0 (+%)	0 (+%)	0 (+%)	0 (+%)	0 (+%)	0 (+%)	0 (+%)	0 (+%)	0 (+%)	0 (+%)	0 (+%)
Faroe Islands	0 (%)	0 (+%)	0 (+%)	0 (+%)	0 (+%)	0 (+%)	0 (+%)	0 (+%)	0 (+%)	0 (+%)	0 (+%)	0 (+%)
Fiji	9 (+56%)	2 (+0%)	2 (+100%)	1 (+100%)	1 (+0%)	0 (%)	1 (+0%)	0 (%)	0 (%)	0 (%)	0 (%)	2 (+50%)
Finland	13 (+1%)	2 (+0%)	3 (+0%)	1 (+100%)	1 (+100%)	0 (%)	1 (+0%)	0 (%)	0 (%)	0 (%)	0 (%)	2 (+0%)
France	681 (+5%)	144 (+3%)	189 (+4%)	80 (+4%)	66 (+6%)	21 (+5%)	32 (+6%)	9 (+0%)	15 (+7%)	10 (+10%)	17 (+6%)	99 (+6%)
French Guia	0 (%)	0 (+%)	0 (+%)	0 (+%)	0 (+%)	0 (+%)	0 (+%)	0 (+%)	0 (+%)	0 (+%)	0 (+%)	0 (+%)
French Polynesia	0 (%)	0 (+%)	0 (+%)	0 (+%)	0 (+%)	0 (+%)	0 (+%)	0 (+%)	0 (+%)	0 (+%)	0 (+%)	0 (+%)
Gabon	3 (+94%)	1 (+0%)	1 (+0%)	0 (%)	0 (%)	0 (%)	0 (%)	0 (%)	0 (%)	0 (%)	0 (%)	1 (+0%)
Gambia	9 (+71%)	2 (+50%)	3 (+67%)	1 (+100%)	1 (+0%)	0 (%)	0 (%)	0 (%)	0 (%)	0 (%)	0 (%)	1 (+100%)
Georgia	18 (+75%)	4 (+75%)	5 (+60%)	2 (+100%)	2 (+50%)	1 (+0%)	1 (+100%)	0 (%)	0 (%)	0 (%)	0 (%)	3 (+67%)
Germany	1527 (+5%)	341 (+5%)	381 (+5%)	157 (+4%)	122 (+7%)	18 (+6%)	7 (+0%)	4 (+25%)	3 (+0%)	8 (+0%)	89 (+9%)	397 (+4%)
Gha	43 (+122%)	12 (+92%)	11 (+109%)	5 (+140%)	4 (+125%)	2 (+50%)	2 (+250%)	0 (%)	1 (+100%)	1 (+0%)	1 (+100%)	5 (+180%)
Gibraltar	0 (%)	0 (+%)	0 (+%)	0 (+%)	0 (+%)	0 (+%)	0 (+%)	0 (+%)	0 (+%)	0 (+%)	0 (+%)	0 (+%)
Greece	86 (+5%)	19 (+0%)	19 (+5%)	10 (+10%)	8 (+12%)	3 (+0%)	9 (+0%)	0 (%)	1 (+100%)	1 (+0%)	2 (+0%)	14 (+7%)
Greenland	0 (%)	0 (+%)	0 (+%)	0 (+%)	0 (+%)	0 (+%)	0 (+%)	0 (+%)	0 (+%)	0 (+%)	0 (+%)	0 (+%)
Greda	0 (%)	0 (%)	0 (%)	0 (%)	0 (%)	0 (%)	0 (%)	0 (%)	0 (%)	0 (%)	0 (%)	0 (%)
Guadeloupe	2 (+22%)	0 (+%)	1 (+%)	0 (+%)	0 (+%)	0 (+%)	0 (+%)	0 (+%)	0 (+%)	0 (+%)	0 (+%)	0 (+%)
Guam	0 (%)	0 (+%)	0 (+%)	0 (+%)	0 (+%)	0 (+%)	0 (+%)	0 (+%)	0 (+%)	0 (+%)	0 (+%)	0 (+%)
Guatemala	109 (+107%)	22 (+91%)	27 (+107%)	12 (+117%)	10 (+110%)	3 (+67%)	9 (+133%)	1 (+200%)	2 (+150%)	0 (%)	2 (+50%)	20 (+120%)
Guinea	79 (+81%)	18 (+83%)	24 (+83%)	10 (+80%)	7 (+86%)	3 (+67%)	2 (+50%)	2 (+50%)	2 (+100%)	0 (%)	1 (+0%)	10 (+80%)
Guinea-Bissau	10 (+66%)	2 (+100%)	3 (+67%)	1 (+100%)	1 (+100%)	0 (%)	0 (%)	0 (%)	0 (%)	0 (%)	0 (%)	1 (+100%)
Guya	7 (+117%)	1 (+0%)	2 (+100%)	1 (+100%)	1 (+100%)	0 (%)	1 (+100%)	0 (%)	0 (%)	0 (%)	0 (%)	2 (+100%)
Haiti	29 (+39%)	8 (+25%)	8 (+38%)	3 (+67%)	3 (+33%)	1 (+0%)	1 (+100%)	1 (+0%)	1 (+0%)	0 (%)	0 (%)	3 (+67%)
Holy See	0 (%)	0 (+%)	0 (+%)	0 (+%)	0 (+%)	0 (+%)	0 (+%)	0 (+%)	0 (+%)	0 (+%)	0 (+%)	0 (+%)
Honduras	59 (+93%)	8 (+75%)	15 (+80%)	7 (+86%)	6 (+83%)	1 (+100%)	7 (+100%)	1 (+0%)	1 (+100%)	0 (%)	1 (+100%)	13 (+100%)
Hungary	176 (+9%)	35 (+9%)	35 (+11%)	19 (+11%)	18 (+11%)	3 (+0%)	22 (+9%)	0 (%)	4 (+0%)	2 (+0%)	3 (+0%)	34 (+9%)
Iceland	0 (%)	0 (%)	0 (%)	0 (%)	0 (%)	0 (%)	0 (%)	0 (%)	0 (%)	0 (%)	0 (%)	0 (%)
India	2633 (+82%)	628 (+41%)	731 (+69%)	315 (+77%)	232 (+92%)	84 (+40%)	119 (+243%)	49 (+29%)	67 (+51%)	9 (+11%)	29 (+159%)	372 (+142%)
Indonesia	784 (+148%)	118 (+119%)	180 (+141%)	89 (+146%)	77 (+153%)	15 (+120%)	95 (+177%)	5 (+80%)	13 (+131%)	3 (+133%)	16 (+162%)	172 (+165%)
Iran (Islamic Republic of)	1807 (+30%)	458 (+%)	493 (+%)	219 (+%)	169 (+%)	78 (+%)	91 (+%)	21 (+%)	28 (+%)	23 (+%)	60 (+%)	165 (+%)
Iraq	83 (+176%)	20 (+%)	21 (+%)	10 (+%)	7 (+%)	3 (+%)	5 (+%)	1 (+%)	2 (+%)	1 (+%)	1 (+%)	11 (+%)
Ireland	100 (+3%)	27 (+4%)	23 (+4%)	11 (+0%)	9 (+0%)	3 (+0%)	7 (+14%)	1 (+0%)	2 (+50%)	1 (+0%)	1 (+0%)	14 (+7%)
Israel	106 (+51%)	11 (+36%)	24 (+46%)	12 (+50%)	11 (+45%)	2 (+0%)	16 (+50%)	0 (%)	1 (+100%)	0 (%)	3 (+33%)	27 (+52%)
Italy	1319 (+6%)	321 (+5%)	268 (+6%)	136 (+7%)	127 (+6%)	25 (+4%)	142 (+8%)	6 (+0%)	29 (+3%)	15 (+0%)	19 (+5%)	230 (+7%)
Jamaica	27 (+50%)	3 (+67%)	6 (+50%)	3 (+67%)	3 (+33%)	0 (%)	4 (+50%)	0 (%)	0 (%)	0 (%)	1 (+0%)	7 (+43%)
Japan	742 (+10%)	322 (+9%)	76 (+16%)	118 (+12%)	102 (+9%)	44 (+14%)	6 (+17%)	4 (+0%)	26 (+12%)	33 (+6%)	12 (+0%)	0 (%)
Jordan	51 (+73%)	7 (+%)	11 (+%)	6 (+%)	5 (+%)	1 (+%)	7 (+%)	0 (+%)	1 (+%)	0 (+%)	1 (+%)	12 (+%)
Kazakhstan	234 (+48%)	62 (+37%)	61 (+48%)	28 (+43%)	21 (+48%)	8 (+25%)	12 (+67%)	4 (+25%)	5 (+60%)	2 (+0%)	3 (+33%)	30 (+70%)
Kenya	395 (+41%)	110 (+41%)	112 (+42%)	47 (+43%)	33 (+42%)	14 (+43%)	9 (+44%)	8 (+50%)	11 (+36%)	2 (+0%)	3 (+67%)	44 (+45%)
Kiribati	0 (%)	0 (+%)	0 (+%)	0 (+%)	0 (+%)	0 (+%)	0 (+%)	0 (+%)	0 (+%)	0 (+%)	0 (+%)	0 (+%)
Kuwait	24 (+84%)	3 (+%)	5 (+%)	3 (+%)	2 (+%)	0 (+%)	3 (+%)	0 (+%)	0 (+%)	0 (+%)	1 (+%)	6 (+%)
Kyrgyzstan	38 (+27%)	11 (+18%)	10 (+30%)	5 (+20%)	3 (+33%)	2 (+0%)	1 (+100%)	1 (+0%)	1 (+0%)	0 (%)	1 (+0%)	4 (+50%)
Lao People's Democratic Republic	34 (+139%)	8 (+%)	9 (+%)	4 (+%)	3 (+%)	1 (+%)	2 (+%)	1 (+%)	1 (+%)	0 (+%)	0 (+%)	5 (+%)
Latvia	6 (+15%)	1 (+0%)	1 (+100%)	1 (+0%)	1 (+0%)	0 (%)	1 (+0%)	0 (%)	0 (%)	0 (%)	0 (%)	1 (+0%)
Lebanon	39 (+63%)	3 (+67%)	9 (+67%)	5 (+60%)	4 (+75%)	1 (+0%)	6 (+67%)	0 (%)	0 (%)	0 (%)	1 (+100%)	10 (+60%)
Lesotho	9 (+2%)	2 (+0%)	2 (+50%)	1 (+0%)	1 (+0%)	0 (%)	0 (%)	0 (%)	0 (%)	0 (%)	0 (%)	1 (+0%)
Liberia	3 (+115%)	1 (+0%)	1 (+0%)	0 (%)	0 (%)	0 (%)	0 (%)	0 (%)	0 (%)	0 (%)	0 (%)	0 (%)
Libya	53 (+68%)	10 (+%)	12 (+%)	6 (+%)	5 (+%)	2 (+%)	6 (+%)	0 (+%)	1 (+%)	1 (+%)	1 (+%)	10 (+%)
Liechtenstein	0 (%)	0 (+%)	0 (+%)	0 (+%)	0 (+%)	0 (+%)	0 (+%)	0 (+%)	0 (+%)	0 (+%)	0 (+%)	0 (+%)
Lithuania	13 (+6%)	2 (+0%)	3 (+0%)	1 (+100%)	1 (+0%)	0 (%)	2 (+0%)	0 (%)	0 (%)	0 (%)	0 (%)	3 (+0%)
Luxembourg	3 (+-4%)	1 (+0%)	1 (+0%)	0 (%)	0 (%)	0 (%)	0 (%)	0 (%)	0 (%)	0 (%)	0 (%)	0 (%)

Madagascar	158 (+49%)	43 (+49%)	45 (+49%)	18 (+50%)	13 (+54%)	5 (+40%)	5 (+20%)	3 (+67%)	5 (+40%)	0 (%)	1 (+100%)	20 (+50%)
Malawi	41 (+71%)	11 (+73%)	11 (+64%)	5 (+60%)	4 (+50%)	1 (+200%)	2 (+100%)	1 (+0%)	1 (+100%)	0 (%)	0 (%)	5 (+60%)
Malaysia	367 (+61%)	40 (+70%)	78 (+63%)	41 (+61%)	37 (+62%)	5 (+80%)	56 (+59%)	1 (+0%)	5 (+60%)	1 (+100%)	9 (+56%)	93 (+59%)
Maldives	0 (%)	0 (+%)	0 (+%)	0 (+%)	0 (+%)	0 (+%)	0 (+%)	0 (+%)	0 (+%)	0 (+%)	0 (+%)	0 (+%)
Mali	150 (+89%)	43 (+86%)	41 (+85%)	19 (+89%)	13 (+92%)	6 (+100%)	4 (+125%)	3 (+67%)	4 (+50%)	2 (+50%)	2 (+100%)	15 (+87%)
Malta	2 (+34%)	0 (%)	1 (+0%)	0 (%)	0 (%)	0 (%)	0 (%)	0 (%)	0 (%)	0 (%)	0 (%)	1 (+0%)
Marshall Islands	0 (%)	0 (+%)	0 (+%)	0 (+%)	0 (+%)	0 (+%)	0 (+%)	0 (+%)	0 (+%)	0 (+%)	0 (+%)	0 (+%)
Martinique	1 (+-75%)	0 (+%)	0 (+%)	0 (+%)	0 (+%)	0 (+%)	0 (+%)	0 (+%)	0 (+%)	0 (+%)	0 (+%)	0 (+%)
Mauritania	45 (+94%)	14 (+%)	12 (+%)	6 (+%)	4 (+%)	2 (+%)	1 (+%)	1 (+%)	1 (+%)	1 (+%)	1 (+%)	3 (+%)
Mauritius	12 (+66%)	1 (+0%)	3 (+33%)	1 (+100%)	1 (+100%)	0 (%)	2 (+50%)	0 (%)	0 (%)	0 (%)	0 (%)	3 (+100%)
Mayotte	0 (%)	0 (+%)	0 (+%)	0 (+%)	0 (+%)	0 (+%)	0 (+%)	0 (+%)	0 (+%)	0 (+%)	0 (+%)	0 (+%)
Mexico	1385 (+65%)	268 (+55%)	316 (+63%)	153 (+64%)	132 (+67%)	28 (+57%)	148 (+76%)	11 (+45%)	27 (+56%)	7 (+57%)	23 (+78%)	272 (+72%)
Micronesia (Federated States of)	0 (%)	0 (+%)	0 (+%)	0 (+%)	0 (+%)	0 (+%)	0 (+%)	0 (+%)	0 (+%)	0 (+%)	0 (+%)	0 (+%)
Moco	0 (%)	0 (+%)	0 (+%)	0 (+%)	0 (+%)	0 (+%)	0 (+%)	0 (+%)	0 (+%)	0 (+%)	0 (+%)	0 (+%)
Mongolia	133 (+2%)	41 (+-2%)	34 (+9%)	17 (+0%)	11 (+9%)	6 (+0%)	4 (+-25%)	2 (+0%)	2 (+50%)	2 (+0%)	2 (+0%)	10 (+20%)
Montenegro	3 (+17%)	1 (+0%)	1 (+0%)	0 (%)	0 (%)	0 (%)	0 (%)	0 (%)	0 (%)	0 (%)	0 (%)	0 (%)
Montserrat	0 (%)	0 (+%)	0 (+%)	0 (+%)	0 (+%)	0 (+%)	0 (+%)	0 (+%)	0 (+%)	0 (+%)	0 (+%)	0 (+%)
Morocco	213 (+74%)	22 (+55%)	53 (+70%)	27 (+70%)	22 (+77%)	6 (+50%)	26 (+88%)	1 (+100%)	3 (+67%)	1 (+100%)	5 (+80%)	46 (+87%)
Mozambique	36 (+91%)	9 (+89%)	9 (+89%)	4 (+100%)	3 (+100%)	1 (+100%)	2 (+100%)	1 (+0%)	1 (+100%)	0 (%)	0 (%)	4 (+125%)
Myanmar	312 (+179%)	69 (+107%)	81 (+154%)	36 (+175%)	29 (+193%)	8 (+112%)	21 (+362%)	5 (+40%)	8 (+112%)	1 (+200%)	4 (+275%)	50 (+262%)
mibia	40 (+65%)	10 (+60%)	11 (+73%)	5 (+60%)	3 (+100%)	1 (+100%)	1 (+100%)	1 (+0%)	1 (+100%)	0 (%)	0 (%)	5 (+60%)
uru	0 (%)	0 (+%)	0 (+%)	0 (+%)	0 (+%)	0 (+%)	0 (+%)	0 (+%)	0 (+%)	0 (+%)	0 (+%)	0 (+%)
Nepal	32 (+42%)	11 (+45%)	8 (+38%)	3 (+67%)	2 (+50%)	1 (+0%)	1 (+100%)	1 (+0%)	1 (+0%)	0 (%)	0 (%)	3 (+100%)
Netherlands	225 (+4%)	89 (+2%)	47 (+4%)	43 (+5%)	31 (+3%)	1 (+0%)	4 (+0%)	0 (%)	5 (+0%)	1 (+0%)	2 (+0%)	3 (+0%)
Netherlands Antilles	0 (%)	0 (+%)	0 (+%)	0 (+%)	0 (+%)	0 (+%)	0 (+%)	0 (+%)	0 (+%)	0 (+%)	0 (+%)	0 (+%)
New Caledonia	1 (+30%)	0 (+%)	0 (+%)	0 (+%)	0 (+%)	0 (+%)	0 (+%)	0 (+%)	0 (+%)	0 (+%)	0 (+%)	0 (+%)
New Zealand	57 (+15%)	7 (+0%)	18 (+6%)	5 (+20%)	5 (+20%)	1 (+0%)	7 (+29%)	1 (+0%)	1 (+0%)	0 (%)	1 (+0%)	12 (+17%)
Nicaragua	76 (+129%)	13 (+115%)	21 (+129%)	9 (+133%)	7 (+143%)	2 (+100%)	5 (+180%)	1 (+200%)	2 (+100%)	0 (%)	1 (+100%)	14 (+136%)
Niger	196 (+89%)	53 (+87%)	57 (+89%)	24 (+88%)	17 (+88%)	7 (+100%)	4 (+100%)	4 (+100%)	5 (+100%)	1 (+100%)	2 (+50%)	22 (+91%)
Nigeria	458 (+96%)	135 (+88%)	117 (+97%)	56 (+95%)	40 (+95%)	18 (+89%)	18 (+122%)	6 (+117%)	10 (+90%)	5 (+100%)	6 (+100%)	46 (+120%)
Niue	0 (%)	0 (+%)	0 (+%)	0 (+%)	0 (+%)	0 (+%)	0 (+%)	0 (+%)	0 (+%)	0 (+%)	0 (+%)	0 (+%)
Northern Maria Islands	0 (%)	0 (+%)	0 (+%)	0 (+%)	0 (+%)	0 (+%)	0 (+%)	0 (+%)	0 (+%)	0 (+%)	0 (+%)	0 (+%)
Norway	7 (+22%)	1 (+0%)	2 (+0%)	1 (+0%)	1 (+0%)	0 (%)	1 (+0%)	0 (%)	0 (%)	0 (%)	0 (%)	1 (+100%)
Occupied Palestinian Territory	5 (+-15%)	2 (+%)	1 (+%)	1 (+%)	0 (+%)	0 (+%)	0 (+%)	0 (+%)	0 (+%)	0 (+%)	0 (+%)	0 (+%)
Oman	22 (+14%)	6 (+%)	6 (+%)	3 (+%)	2 (+%)	1 (+%)	1 (+%)	0 (+%)	0 (+%)	0 (+%)	0 (+%)	2 (+%)
Pakistan	1031 (+81%)	245 (+%)	283 (+%)	125 (+%)	92 (+%)	35 (+%)	49 (+%)	18 (+%)	25 (+%)	5 (+%)	13 (+%)	142 (+%)
Palau	0 (%)	0 (+%)	0 (+%)	0 (+%)	0 (+%)	0 (+%)	0 (+%)	0 (+%)	0 (+%)	0 (+%)	0 (+%)	0 (+%)
Pama	54 (+72%)	8 (+%)	13 (+%)	6 (+%)	5 (+%)	1 (+%)	6 (+%)	0 (+%)	1 (+%)	0 (+%)	1 (+%)	12 (+%)
Papua New Guinea	10 (+76%)	2 (+50%)	2 (+100%)	1 (+100%)	1 (+100%)	0 (%)	1 (+100%)	0 (%)	0 (%)	0 (%)	0 (%)	2 (+50%)
Paraguay	221 (+55%)	60 (+52%)	62 (+53%)	25 (+56%)	19 (+58%)	7 (+43%)	6 (+100%)	5 (+40%)	7 (+43%)	0 (%)	1 (+100%)	29 (+59%)
Peru	364 (+114%)	48 (+88%)	83 (+110%)	42 (+110%)	36 (+119%)	7 (+86%)	48 (+129%)	2 (+50%)	5 (+120%)	1 (+100%)	8 (+125%)	84 (+126%)
Philippines	316 (+194%)	61 (+169%)	69 (+183%)	35 (+189%)	32 (+194%)	6 (+167%)	37 (+222%)	2 (+50%)	6 (+200%)	3 (+200%)	5 (+220%)	61 (+213%)
Poland	576 (+9%)	101 (+8%)	122 (+8%)	63 (+8%)	60 (+8%)	10 (+10%)	73 (+11%)	2 (+0%)	12 (+8%)	6 (+17%)	10 (+10%)	117 (+9%)
Portugal	179 (+9%)	36 (+8%)	38 (+11%)	19 (+11%)	18 (+6%)	3 (+33%)	21 (+10%)	1 (+0%)	4 (+0%)	2 (+0%)	3 (+0%)	35 (+9%)
Puerto Rico	25 (+-59%)	4 (+%)	6 (+%)	3 (+%)	2 (+%)	0 (+%)	3 (+%)	0 (+%)	0 (+%)	0 (+%)	0 (+%)	5 (+%)
Qatar	6 (+69%)	1 (+%)	1 (+%)	1 (+%)	1 (+%)	0 (+%)	1 (+%)	0 (+%)	0 (+%)	0 (+%)	0 (+%)	1 (+%)
Reunion	7 (+-89%)	1 (+%)	2 (+%)	1 (+%)	1 (+%)	0 (+%)	1 (+%)	0 (+%)	0 (+%)	0 (+%)	0 (+%)	2 (+%)
Republic of Korea	938 (+42%)	156 (+42%)	241 (+37%)	132 (+41%)	75 (+37%)	57 (+39%)	60 (+63%)	11 (+45%)	115 (+39%)	25 (+36%)	13 (+38%)	53 (+58%)
Republic of Moldova	13 (+92%)	2 (+%)	3 (+%)	1 (+%)	1 (+%)	0 (+%)	2 (+%)	0 (+%)	0 (+%)	0 (+%)	0 (+%)	3 (+%)
Romania	242 (+9%)	52 (+8%)	54 (+7%)	27 (+7%)	24 (+8%)	6 (+0%)	25 (+12%)	2 (+0%)	5 (+20%)	3 (+0%)	4 (+0%)	42 (+12%)
Russian Federation	1581 (+17%)	304 (+10%)	337 (+17%)	172 (+17%)	156 (+18%)	30 (+10%)	189 (+23%)	7 (+0%)	30 (+10%)	13 (+8%)	28 (+25%)	314 (+22%)
Rwanda	22 (+60%)	6 (+50%)	6 (+67%)	3 (+33%)	2 (+50%)	1 (+0%)	1 (+0%)	0 (%)	1 (+0%)	0 (%)	0 (%)	2 (+100%)
"Saint Hele, Ascension and Tristan da Cunha"	0 (+%)	0 (%)	0 (+%)	0 (+%)	0 (+%)	0 (+%)	0 (+%)	0 (+%)	0 (+%)	0 (+%)	0 (+%)	0 (+%)
Saint Kitts and Nevis	3 (+58%)	0 (%)	1 (+0%)	0 (%)	0 (%)	0 (%)	0 (%)	0 (%)	0 (%)	0 (%)	0 (%)	1 (+0%)
Saint Lucia	1 (+34%)	0 (%)	0 (%)	0 (%)	0 (%)	0 (%)	0 (%)	0 (%)	0 (%)	0 (%)	0 (%)	0 (%)
Saint Pierre and Miquelon	0 (%)	0 (+%)	0 (+%)	0 (+%)	0 (+%)	0 (+%)	0 (+%)	0 (+%)	0 (+%)	0 (+%)	0 (+%)	0 (+%)
Saint Vincent and the Gredines	0 (%)	0 (%)	0 (%)	0 (%)	0 (%)	0 (%)	0 (%)	0 (%)	0 (%)	0 (%)	0 (%)	0 (%)
Samoa	1 (+74%)	0 (+%)	0 (+%)	0 (+%)	0 (+%)	0 (+%)	0 (+%)	0 (+%)	0 (+%)	0 (+%)	0 (+%)	0 (+%)
San Marino	0 (%)	0 (+%)	0 (+%)	0 (+%)	0 (+%)	0 (+%)	0 (+%)	0 (+%)	0 (+%)	0 (+%)	0 (+%)	0 (+%)
Sao Tome and Principe	1 (+30%)	0 (%)	0 (%)	0 (%)	0 (%)	0 (%)	0 (%)	0 (%)	0 (%)	0 (%)	0 (%)	0 (%)
Saudi Arabia	182 (+72%)	26 (+%)	40 (+%)	21 (+%)	18 (+%)	4 (+%)	24 (+%)	1 (+%)	2 (+%)	1 (+%)	5 (+%)	41 (+%)

Senegal	70 (+77%)	16 (+69%)	19 (+74%)	9 (+67%)	6 (+83%)	2 (+100%)	3 (+133%)	1 (+100%)	2 (+50%)	0 (%)	1 (+100%)	9 (+100%)
Serbia	87 (+10%)	19 (+11%)	19 (+11%)	10 (+10%)	9 (+11%)	2 (+0%)	9 (+11%)	0 (%)	2 (+0%)	1 (+0%)	1 (+0%)	15 (+7%)
Seychelles	0 (%)	0 (+%)	0 (+%)	0 (+%)	0 (+%)	0 (+%)	0 (+%)	0 (+%)	0 (+%)	0 (+%)	0 (+%)	0 (+%)
Sierra Leone	4 (+67%)	1 (+%)	1 (+%)	1 (+%)	0 (+%)	0 (+%)	0 (+%)	0 (+%)	0 (+%)	0 (+%)	0 (+%)	1 (+%)
Singapore	23 (+95%)	3 (+%)	5 (+%)	3 (+%)	2 (+%)	0 (+%)	4 (+%)	0 (+%)	0 (+%)	0 (+%)	1 (+%)	6 (+%)
Slovakia	16 (+6%)	3 (+0%)	4 (+0%)	2 (+0%)	2 (+0%)	0 (%)	2 (+0%)	0 (%)	0 (%)	0 (%)	0 (%)	3 (+0%)
Slovenia	4 (+9%)	0 (%)	1 (+0%)	0 (%)	0 (%)	0 (%)	1 (+0%)	0 (%)	0 (%)	0 (%)	0 (%)	1 (+0%)
Solomon Islands	1 (+3%)	0 (+%)	0 (+%)	0 (+%)	0 (+%)	0 (+%)	0 (+%)	0 (+%)	0 (+%)	0 (+%)	0 (+%)	0 (+%)
Somalia	126 (+99%)	30 (+%)	37 (+%)	17 (+%)	11 (+%)	5 (+%)	3 (+%)	3 (+%)	3 (+%)	1 (+%)	1 (+%)	14 (+%)
South Africa	729 (+19%)	144 (+11%)	181 (+17%)	85 (+18%)	68 (+21%)	19 (+11%)	64 (+30%)	8 (+12%)	14 (+14%)	3 (+0%)	12 (+25%)	132 (+26%)
Spain	2202 (+6%)	560 (+5%)	433 (+6%)	226 (+6%)	217 (+6%)	43 (+5%)	240 (+8%)	7 (+0%)	51 (+6%)	31 (+6%)	29 (+7%)	365 (+7%)
Sri Lanka	20 (+86%)	3 (+67%)	5 (+60%)	2 (+100%)	2 (+100%)	0 (%)	2 (+150%)	0 (%)	0 (%)	0 (%)	0 (%)	5 (+80%)
Sudan (former)	2 (+-100%)	0 (+%)	0 (+%)	0 (+%)	0 (+%)	0 (+%)	0 (+%)	0 (+%)	0 (+%)	0 (+%)	0 (+%)	1 (+%)
Surime	4 (+41%)	0 (%)	1 (+0%)	0 (%)	0 (%)	0 (%)	1 (+0%)	0 (%)	0 (%)	0 (%)	0 (%)	1 (+0%)
Swaziland	8 (+20%)	2 (+0%)	2 (+50%)	1 (+0%)	1 (+0%)	0 (%)	0 (%)	0 (%)	0 (%)	0 (%)	0 (%)	1 (+100%)
Sweden	10 (+7%)	1 (+0%)	3 (+0%)	1 (+0%)	1 (+0%)	0 (%)	1 (+0%)	0 (%)	0 (%)	0 (%)	0 (%)	2 (+0%)
Switzerland	49 (+-3%)	11 (+-9%)	11 (+0%)	5 (+0%)	5 (+0%)	1 (+0%)	5 (+0%)	0 (%)	1 (+0%)	1 (+0%)	1 (+0%)	8 (+0%)
Syrian Arab Republic	135 (+15%)	39 (+%)	32 (+%)	17 (+%)	12 (+%)	6 (+%)	7 (+%)	1 (+%)	2 (+%)	2 (+%)	3 (+%)	13 (+%)
Tajikistan	49 (+47%)	6 (+33%)	17 (+53%)	7 (+43%)	5 (+40%)	2 (+50%)	1 (+100%)	1 (+100%)	2 (+50%)	0 (%)	0 (%)	7 (+57%)
Thailand	531 (+68%)	91 (+65%)	114 (+67%)	58 (+67%)	53 (+70%)	9 (+67%)	67 (+75%)	2 (+50%)	10 (+60%)	4 (+50%)	10 (+70%)	113 (+71%)
The former Yugoslav Republic of Macedonia	1 (+0%)	7 (+5%)	2 (+0%)	2 (+0%)	1 (+0%)	1 (+0%)	0 (%)	0 (%)	0 (%)	0 (%)	0 (%)	0 (%)
Timor-Leste	3 (+145%)	1 (+100%)	1 (+100%)	0 (%)	0 (%)	0 (%)	0 (%)	0 (%)	0 (%)	0 (%)	0 (%)	0 (%)
Togo	21 (+79%)	5 (+60%)	6 (+67%)	3 (+67%)	2 (+50%)	1 (+0%)	1 (+100%)	0 (%)	0 (%)	0 (%)	0 (%)	3 (+67%)
Tokelau	0 (%)	0 (+%)	0 (+%)	0 (+%)	0 (+%)	0 (+%)	0 (+%)	0 (+%)	0 (+%)	0 (+%)	0 (+%)	0 (+%)
Tonga	0 (%)	0 (+%)	0 (+%)	0 (+%)	0 (+%)	0 (+%)	0 (+%)	0 (+%)	0 (+%)	0 (+%)	0 (+%)	0 (+%)
Trinidad and Tobago	24 (+28%)	2 (+0%)	5 (+40%)	3 (+0%)	3 (+0%)	0 (%)	4 (+25%)	0 (%)	0 (%)	0 (%)	1 (+0%)	7 (+29%)
Tunisia	72 (+67%)	8 (+50%)	18 (+61%)	9 (+67%)	7 (+71%)	2 (+50%)	9 (+78%)	0 (%)	1 (+100%)	0 (%)	2 (+50%)	16 (+75%)
Turkey	658 (+70%)	107 (+%)	157 (+%)	77 (+%)	63 (+%)	16 (+%)	72 (+%)	5 (+%)	10 (+%)	3 (+%)	14 (+%)	135 (+%)
Turkmenistan	92 (+22%)	24 (+0%)	25 (+28%)	12 (+17%)	8 (+25%)	4 (+0%)	3 (+67%)	1 (+100%)	2 (+50%)	1 (+0%)	1 (+0%)	10 (+50%)
Turks and Caicos Islands	0 (%)	0 (+%)	0 (+%)	0 (+%)	0 (+%)	0 (+%)	0 (+%)	0 (+%)	0 (+%)	0 (+%)	0 (+%)	0 (+%)
Tuvalu	0 (%)	0 (+%)	0 (+%)	0 (+%)	0 (+%)	0 (+%)	0 (+%)	0 (+%)	0 (+%)	0 (+%)	0 (+%)	0 (+%)
Uganda	199 (+100%)	55 (+93%)	56 (+96%)	24 (+96%)	17 (+100%)	7 (+86%)	6 (+133%)	4 (+100%)	5 (+120%)	1 (+100%)	2 (+100%)	23 (+117%)
Ukraine	422 (+26%)	69 (+26%)	92 (+24%)	46 (+26%)	42 (+29%)	7 (+29%)	54 (+28%)	2 (+0%)	8 (+25%)	3 (+33%)	8 (+25%)	91 (+25%)
United Arab Emirates	26 (+43%)	6 (+%)	6 (+%)	3 (+%)	2 (+%)	1 (+%)	3 (+%)	0 (+%)	0 (+%)	0 (+%)	1 (+%)	4 (+%)
United Kingdom	422 (+5%)	89 (+3%)	105 (+5%)	51 (+6%)	46 (+7%)	13 (+8%)	26 (+8%)	2 (+0%)	8 (+0%)	6 (+0%)	16 (+6%)	60 (+7%)
United Republic of Tanzania	583 (+57%)	148 (+55%)	175 (+56%)	70 (+59%)	50 (+58%)	20 (+55%)	11 (+100%)	14 (+50%)	18 (+50%)	1 (+100%)	4 (+50%)	72 (+62%)
United States of America	9476 (+22%)	1814 (+15%)	2056 (+21%)	1021 (+22%)	919 (+23%)	170 (+16%)	1112 (+28%)	54 (+7%)	180 (+15%)	58 (+12%)	164 (+29%)	1929 (+26%)
United States Virgin Islands	0 (%)	0 (+%)	0 (+%)	0 (+%)	0 (+%)	0 (+%)	0 (+%)	0 (+%)	0 (+%)	0 (+%)	0 (+%)	0 (+%)
Uruguay	322 (+11%)	85 (+11%)	94 (+11%)	38 (+11%)	27 (+11%)	10 (+10%)	8 (+12%)	7 (+14%)	10 (+10%)	0 (%)	2 (+0%)	41 (+12%)
Uzbekistan	216 (+54%)	51 (+45%)	64 (+55%)	27 (+52%)	19 (+58%)	8 (+38%)	6 (+83%)	5 (+40%)	6 (+67%)	1 (+0%)	2 (+50%)	28 (+64%)
Vanuatu	3 (+29%)	1 (+%)	1 (+%)	0 (+%)	0 (+%)	0 (+%)	0 (+%)	0 (+%)	0 (+%)	0 (+%)	0 (+%)	0 (+%)
Venezuela (Bolivarian Republic of)	568 (+58%)	104 (+48%)	138 (+56%)	64 (+58%)	54 (+57%)	12 (+50%)	56 (+71%)	6 (+50%)	12 (+42%)	1 (+100%)	9 (+78%)	112 (+67%)
Viet m	515 (+215%)	79 (+%)	112 (+%)	44 (+%)	42 (+%)	7 (+%)	35 (+%)	3 (+%)	9 (+%)	4 (+%)	2 (+%)	176 (+%)
Wallis and Futu Islands	0 (%)	0 (+%)	0 (+%)	0 (+%)	0 (+%)	0 (+%)	0 (+%)	0 (+%)	0 (+%)	0 (+%)	0 (+%)	0 (+%)
Western Sahara	0 (%)	0 (+%)	0 (+%)	0 (+%)	0 (+%)	0 (+%)	0 (+%)	0 (+%)	0 (+%)	0 (+%)	0 (+%)	0 (+%)
Yemen	94 (+58%)	21 (+%)	23 (+%)	12 (+%)	9 (+%)	3 (+%)	7 (+%)	1 (+%)	2 (+%)	1 (+%)	2 (+%)	14 (+%)
Zambia	43 (+65%)	10 (+60%)	12 (+58%)	5 (+60%)	4 (+50%)	1 (+100%)	2 (+100%)	1 (+0%)	1 (+100%)	0 (%)	0 (%)	6 (+67%)
Zimbabwe	71 (+16%)	19 (+11%)	20 (+15%)	8 (+25%)	6 (+17%)	2 (+50%)	3 (+0%)	1 (+100%)	2 (+0%)	0 (%)	1 (+0%)	9 (+22%)

Table S3. Retail prices of antimicrobials per kilogram of active ingredient in 2016 ($US).

Country	Tetracyclines	Amphenicols	Penicillins	Cephalosporins	Sulfonamides	Macrolides	Aminoglycosides	Quinolones	Pleuromutilins	Polymyxins	Sources
Argentina	308		544	2620		437	9				Tecnofarm
Argentina			658								Tecnofarm
Argentina			330								Tecnofarm
Australia	2600										http://hobsonandco.co.za/veterinary-products/; www.petceutics.com.au
Australia	18500										http://hobsonandco.co.za/veterinary-products/; www.petceutics.com.au
Australia	1767										http://hobsonandco.co.za/veterinary-products/; www.petceutics.com.au
Australia	771		1300								http://hobsonandco.co.za/veterinary-products/; www.petceutics.com.au
Australia	1054										http://hobsonandco.co.za/veterinary-products/; www.petceutics.com.au
Bangladesh	18			46		29	29				Samiul Haque
Bangladesh	9										Samiul Haque
Burkino Faso											Veterinarians without Borders
Burkino Faso											Veterinarians without Borders
Burkino Faso											Veterinarians without Borders
Burkino Faso						500	2525				Veterinarians without Borders
Burkino Faso											Veterinarians without Borders
Burkino Faso											Veterinarians without Borders
Burkino Faso											Veterinarians without Borders
Burkino Faso											Veterinarians without Borders
Canada	421		280		48						http://www.petware.ca/pet_detail.cfm?petid=5&id=18473
Canada					240						http://www.petware.ca/pet_detail.cfm?petid=5&id=18474
Canada					1154						http://www.petware.ca/pet_detail.cfm?petid=5&id=18475
Chile	637		706	6230	179	31272	285	9393			Juan Josâ Cifuentes Fernendez por Copeval
Chile	568		207	22204	233	736	418	1334			Juan Josâ Cifuentes Fernendez por Copeval
Chile	1493		78	869	27		15764	214			Juan Josâ Cifuentes Fernendez por Copeval
Chile	1204		374	8056	835		10590	1944			Juan Josâ Cifuentes Fernendez por Copeval
Chile	336		236	10558	503		756	2834			Juan Josâ Cifuentes Fernendez por Copeval
Chile	856		864	6420	1568		880	1185333			Juan Josâ Cifuentes Fernendez por Copeval
Chile	482		324		621		1468	6124			Juan Josâ Cifuentes Fernendez por Copeval
Chile	897		147		541		196	10430			Juan Josâ Cifuentes Fernendez por Copeval
Chile			124		552			2820			Juan Josâ Cifuentes Fernendez por Copeval
Chile			1543					22390			Juan Josâ Cifuentes Fernendez por Copeval
Chile			700								Juan Josâ Cifuentes Fernendez por Copeval
Chile			1546								Juan Josâ Cifuentes Fernendez por Copeval
Chile			701								Juan Josâ Cifuentes Fernendez por Copeval
Chile			5053								Juan Josâ Cifuentes Fernendez por Copeval
Chile			1854								Juan Josâ Cifuentes Fernendez por Copeval
Chile			5118								Juan Josâ Cifuentes Fernendez por Copeval
Chile			1878								Juan Josâ Cifuentes Fernendez por Copeval
Chile			1840								Juan Josâ Cifuentes Fernendez por Copeval
China	1573		112			6820		5667		442	Yolanda; https://tianyuan-pharm.en.alibaba.com/
Costa Rica	825			4800				3300			Victor Sanchez from Navet SA
Costa Rica	670							800			Victor Sanchez from Navet SA
Costa Rica	493							200			Victor Sanchez from Navet SA
Costa Rica	400										Victor Sanchez from Navet SA
Kenya	226		366			240	190				Sara Imbach; VSF Switzerland
Kenya	345										Sara Imbach; VSF Switzerland
Kenya	235										Sara Imbach; VSF Switzerland
Kenya	135										Sara Imbach; VSF Switzerland
Kenya	58										Sara Imbach; VSF Switzerland
Kenya	172										Sara Imbach; VSF Switzerland
Kenya	157		385								Sara Imbach; VSF Switzerland
Kenya	150		986								Sara Imbach; VSF Switzerland
Kenya	165		949								Sara Imbach; VSF Switzerland
Kenya	196		1373								Sara Imbach; VSF Switzerland
Kenya	582		2690								Sara Imbach; VSF Switzerland
Kenya	388										Sara Imbach; VSF Switzerland
Kenya	1		67		7	19	642	10618			Sara Imbach; VSF Switzerland
Kenya	1		411				22	8625			Sara Imbach; VSF Switzerland
Kenya	3		2				22				Sara Imbach; VSF Switzerland
Kenya	4		0								Sara Imbach; VSF Switzerland
Kenya	1		2								Sara Imbach; VSF Switzerland
Kenya	1		0								Sara Imbach; VSF Switzerland
Kenya	3		4								Sara Imbach; VSF Switzerland
Kenya	6		2								Sara Imbach; VSF Switzerland
Kenya	1		6								Sara Imbach; VSF Switzerland
Kenya	2		23								Sara Imbach; VSF Switzerland
Kenya	4										Sara Imbach; VSF Switzerland
Kenya	8										Sara Imbach; VSF Switzerland
Kenya	1										Sara Imbach; VSF Switzerland
Kenya	2										Sara Imbach; VSF Switzerland
Kenya	4										Sara Imbach; VSF Switzerland
Kenya	6										Sara Imbach; VSF Switzerland
Kenya	3										Sara Imbach; VSF Switzerland
Kenya	3										Sara Imbach; VSF Switzerland
Kenya	4										Sara Imbach; VSF Switzerland
Kenya	4										Sara Imbach; VSF Switzerland
Kenya	7										Sara Imbach; VSF Switzerland
Kenya	17										Sara Imbach; VSF Switzerland
Kenya	4										Sara Imbach; VSF Switzerland
Kenya	14										Sara Imbach; VSF Switzerland
Kenya	2										Sara Imbach; VSF Switzerland
Mexico	15220		5623	63500			24800	21646			http://www.lavet.com.mx/producto-veterinario/
Mexico							7100	14213			http://www.lavet.com.mx/producto-veterinario/
Mexico								25700			http://www.lavet.com.mx/producto-veterinario/
Nepal	140	4681	118	1123	8425	178		187	385		Sharada Thapaliya; Agriculture & Forestry University

Nepal						585		159			Sharada Thapaliya; Agriculture & Forestry University
Niger	167		287		62		412				Veterinarians without Borders
Niger	135		126								Veterinarians without Borders
Niger	254		456								Veterinarians without Borders
Niger	472										Veterinarians without Borders
Niger	236										Veterinarians without Borders
Russia			242	35207		103	311	490			http://vetmarket.ru/catalog/antibakterialnye_preparaty/
Russia			2475	14886		56	168	331			http://vetmarket.ru/catalog/antibakterialnye_preparaty/
Russia			2957	15974		4	12	1267			http://vetmarket.ru/catalog/antibakterialnye_preparaty/
Russia				151040		1024	121	701			http://vetmarket.ru/catalog/antibakterialnye_preparaty/
Russia				147200		294		217			http://vetmarket.ru/catalog/antibakterialnye_preparaty/
Russia						552		1472			http://vetmarket.ru/catalog/antibakterialnye_preparaty/
Russia						710					http://vetmarket.ru/catalog/antibakterialnye_preparaty/
Russia						1691					http://vetmarket.ru/catalog/antibakterialnye_preparaty/
Rwanda	166		222				222				Veterinarians without Borders
Rwanda	332										Veterinarians without Borders
Rwanda	222										Veterinarians without Borders
South Africa	796				41						https://www.vetproductsonline.co.za/products/cattle/antibiotics
South Africa	529				206						https://www.vetproductsonline.co.za/products/cattle/antibiotics
South Africa	4970				34						https://www.vetproductsonline.co.za/products/cattle/antibiotics
South Africa	1303				38						https://www.vetproductsonline.co.za/products/cattle/antibiotics
South Africa	2084				189						https://www.vetproductsonline.co.za/products/cattle/antibiotics
South Africa	495				455						https://www.vetproductsonline.co.za/products/cattle/antibiotics
South Africa	281				7						https://www.vetproductsonline.co.za/products/cattle/antibiotics
South Africa	662				60						https://www.vetproductsonline.co.za/products/cattle/antibiotics
South Africa	375				206						https://www.vetproductsonline.co.za/products/cattle/antibiotics
South Africa	447				258						https://www.vetproductsonline.co.za/products/cattle/antibiotics
South Africa	263				169						https://www.vetproductsonline.co.za/products/cattle/antibiotics
South Africa	525				189						https://www.vetproductsonline.co.za/products/cattle/antibiotics
South Africa	274				227						https://www.vetproductsonline.co.za/products/cattle/antibiotics
South Africa	297				364						https://www.vetproductsonline.co.za/products/cattle/antibiotics
South Africa	602				399						https://www.vetproductsonline.co.za/products/cattle/antibiotics
South Africa	467				300						https://www.vetproductsonline.co.za/products/cattle/antibiotics
South Africa	1230				331						https://www.vetproductsonline.co.za/products/cattle/antibiotics
South Africa	920				495						https://www.vetproductsonline.co.za/products/cattle/antibiotics
South Africa	630				269						https://www.vetproductsonline.co.za/products/cattle/antibiotics
South Africa	364				413						https://www.vetproductsonline.co.za/products/cattle/antibiotics
South Africa	230				185						https://www.vetproductsonline.co.za/products/cattle/antibiotics
South Africa	569				254						https://www.vetproductsonline.co.za/products/cattle/antibiotics
South Africa	371										https://www.vetproductsonline.co.za/products/cattle/antibiotics
South Africa	955										https://www.vetproductsonline.co.za/products/cattle/antibiotics
South Africa	842										https://www.vetproductsonline.co.za/products/cattle/antibiotics
South Africa	580										https://www.vetproductsonline.co.za/products/cattle/antibiotics
South Africa	787										https://www.vetproductsonline.co.za/products/cattle/antibiotics
South Africa	1052										https://www.vetproductsonline.co.za/products/cattle/antibiotics
Spain	1376		193	3081			195	134			http://www.syva.es/pdfs/p_2010-12-13_1292232135.pdf
Spain	1234		1723	17076			172				http://www.syva.es/pdfs/p_2010-12-13_1292232135.pdf
Spain			1312	11511							http://www.syva.es/pdfs/p_2010-12-13_1292232135.pdf
Spain			336								http://www.syva.es/pdfs/p_2010-12-13_1292232135.pdf
Spain			170								http://www.syva.es/pdfs/p_2010-12-13_1292232135.pdf
Spain			3391								http://www.syva.es/pdfs/p_2010-12-13_1292232135.pdf
Uganda	272		27		92		34				http://www.lrrd.org/lrrd23/7/vudr23162.htm; http://www.norbrook.com/products/
Uganda	159										http://www.lrrd.org/lrrd23/7/vudr23162.htm; http://www.norbrook.com/products/
Uganda	136										http://www.lrrd.org/lrrd23/7/vudr23162.htm; http://www.norbrook.com/products/
United Kingdom	480	1425	512	9835	83	1906	41227	6313			farmacy.co.uk
United Kingdom	739	884	352	6061	102	9009		5577			farmacy.co.uk
United Kingdom	675	808	1523	7576	79	14459		5100			farmacy.co.uk
United Kingdom	668	927	721	22525	413	13146		9848			farmacy.co.uk
United Kingdom	582	876	439	22334	512	1065		5682			farmacy.co.uk
United Kingdom	771	14428	367	7164	395	23326		5758			farmacy.co.uk
United Kingdom	658	27399	863	7548		20902		7105			farmacy.co.uk
United Kingdom	745	1011	1022	5620		20100		4125			farmacy.co.uk
United Kingdom	542		757	9098				5528			farmacy.co.uk
United Kingdom	385		754	1408				5179			farmacy.co.uk
United Kingdom	257		674	3725							farmacy.co.uk
United Kingdom	377		1661	316212							farmacy.co.uk
United Kingdom	364		1371	203295							farmacy.co.uk
United Kingdom			674	12233							farmacy.co.uk
United Kingdom			181								farmacy.co.uk
United Kingdom			884								farmacy.co.uk
United States	68				52	161	590				www.valleyvet.com; www.qcsupply.com;
United States	137				"1,760"	117	590				www.valleyvet.com; www.qcsupply.com;
United States	75				129	269	62				www.valleyvet.com; www.qcsupply.com;
United States	39						1283				www.valleyvet.com; www.qcsupply.com;
United States	49										www.valleyvet.com; www.qcsupply.com;
United States	136										www.valleyvet.com; www.qcsupply.com;
United States	69										www.valleyvet.com; www.qcsupply.com;
United States					52	4900					www.valleyvet.com; www.qcsupply.com;
United States					"1,760"						www.valleyvet.com; www.qcsupply.com;
United States					129						www.valleyvet.com; www.qcsupply.com;
United States	30	100	56	505		4900	126	267	1667	30	www.valleyvet.com; www.qcsupply.com;
United States	500			1888		9694	83	106	400		www.valleyvet.com; www.qcsupply.com;
United States	200					3	258				www.valleyvet.com; www.qcsupply.com;
Vietnam	27	49	12	94	26	81	16	21	33	1	http://gauvang.com.vn/tin-tuc/bang-gia-thuoc-thu-y-c01-16195.html
Vietnam	43		20	932	21	59	31	35			http://gauvang.com.vn/tin-tuc/bang-gia-thuoc-thu-y-c01-16195.html
Vietnam	1			195	43	48	6	38			http://gauvang.com.vn/tin-tuc/bang-gia-thuoc-thu-y-c01-16195.html
Vietnam	21			42	49	141	6	759			http://gauvang.com.vn/tin-tuc/bang-gia-thuoc-thu-y-c01-16195.html
Vietnam					10	1000	20				http://gauvang.com.vn/tin-tuc/bang-gia-thuoc-thu-y-c01-16195.html
Vietnam					1	42	100	22			http://gauvang.com.vn/tin-tuc/bang-gia-thuoc-thu-y-c01-16195.html
Vietnam					11	5					http://gauvang.com.vn/tin-tuc/bang-gia-thuoc-thu-y-c01-16195.html
Vietnam					39	32					http://gauvang.com.vn/tin-tuc/bang-gia-thuoc-thu-y-c01-16195.html
Vietnam					27						http://gauvang.com.vn/tin-tuc/bang-gia-thuoc-thu-y-c01-16195.html
Vietnam					19						http://gauvang.com.vn/tin-tuc/bang-gia-thuoc-thu-y-c01-16195.html

Vietnam	14	http://gauvang.com.vn/tin-tuc/bang-gia-thuoc-thu-y-c01-16195.html
Vietnam	25	http://gauvang.com.vn/tin-tuc/bang-gia-thuoc-thu-y-c01-16195.html
Vietnam	10	http://gauvang.com.vn/tin-tuc/bang-gia-thuoc-thu-y-c01-16195.html
Vietnam	6	http://gauvang.com.vn/tin-tuc/bang-gia-thuoc-thu-y-c01-16195.html
Vietnam	6	http://gauvang.com.vn/tin-tuc/bang-gia-thuoc-thu-y-c01-16195.html
Vietnam	11	http://gauvang.com.vn/tin-tuc/bang-gia-thuoc-thu-y-c01-16195.html
Vietnam	18	http://gauvang.com.vn/tin-tuc/bang-gia-thuoc-thu-y-c01-16195.html

References

1. T. P. Van Boeckel *et al.*, Global trends in antimicrobial use in food animals. *Proc. Natl. Acad. Sci.* **112**, 5649–5654 (2015).

2. M. Herrero *et al.*, Biomass use, production, feed efficiencies, and greenhouse gas emissions from global livestock systems. *Proc. Natl. Acad. Sci. U. S. A.* **110**, 20888–20893 (2013).

3. T. P. Robinson *et al.*, Mapping the Global Distribution of Livestock. *PLoS ONE*. **9**, e96084 (2014).

4. P. D. Warriss, *Meat science: an introductory text* (Cabi, 2010; http://books.google.com/books?hl=en&lr=&id=ExEOboVw_KUC&oi=fnd&pg=PP6&dq=Warriss,+P.D.+2010+Meat+Science:+an+introductory+text&ots=p5qKmpcO3J&sig=mKv0xmKBW0rhimOXyD_8pRO6k3s).

5. T. P. Robinson, F. Pozzi, Mapping supply and demand for animal-source foods to 2030. *Anim. Prod. Health Work. Pap.*, 164 (2011).

6. M. Gilbert *et al.*, Income Disparities and the Global Distribution of Intensively Farmed Chicken and Pigs. *PLOS ONE*. **10**, e0133381 (2015).

7. J. O'Neill, Tackling drug-resistant infections globally: Final report and Recommandations (2016), (available at http://amr-review.org/sites/default/files/160525_Final%20paper_with%20cover.pdf).

8. Y. Yang, X. Yang, F. Zhai, Y. Cheng, Dietary Guidelines for Chinese (2016). *J. Acad. Nutr. Diet.* **116**, A37 (2016).

9. S. W. Page, P. Gautier, Use of antimicrobial agents in livestock. *Rev. Sci. Tech.-OIE*. **31**, 145 (2012).
